# Design of Highly Efficient CO_2_-Switchable Solvents Based on Extraction and Recovery Performance

**DOI:** 10.3390/molecules31193444

**Published:** 2026-09-27

**Authors:** Wen Ren, Luanxiao Wei, Yisu Fang, Xingchun Li, Yu Li, Chunmao Chen

**Affiliations:** 1 State Key Laboratory of Petroleum Pollution Control, China University of Petroleum-Beijing, Beijing 102200, China; renwen@cnpc.com.cn (W.R.); c.chen@cup.edu.cn (C.C.); 2 State Key Laboratory of Petroleum Pollution Control, CNPC Research Institute of Safety and Environment Technology, Beijing 102206, China; li-xingchun@cnpc.com.cn; 3 College of Environmental Science and Engineering, North China Electric Power University, Beijing 102206, China; wlxiao2024@163.com (L.W.); 120242232021@ncepu.edu.cn (Y.F.)

**Keywords:** CO_2_-switchable solvents, structural modification, density functional theory, machine learning

## Abstract

CO_2_-switchable solvents (CSSs) show promise for oil-based drilling cuttings treatment and green extraction, yet their structure–efficiency relationships remain unclear. Here, 15 amine-based reference compounds were used to design 163 candidates through C8-equivalent hydrophobic tail standardization and functionalization at the 2C, 3C, and 4C positions of the hydrophobic linker. Density functional theory calculations identified 23 CSSs with improved extraction efficiency, including 14 highly efficient and nine effective candidates. Linear mixed-effects analysis revealed that extraction efficiency was jointly governed by modification site, functional group, and hydrophobic molecular background, with the 3C and 4C positions showing the highest sensitivity. Thermodynamic evaluation indicated that 87% of the selected CSSs combined enhanced extraction with favorable recovery-related thermodynamic characteristics in water and ethanol model environments. Support vector regression coupled with principal component analysis further identified steric effects, polarizability, and electron-donating ability as key structural factors governing efficiency changes. Toxicity prediction using ADMETlab 3.0 showed that hydroxyl and ester modifications generally reduced hepatotoxicity, nephrotoxicity, and aquatic toxicity. Overall, this study provides mechanistic insights and design guidelines for developing efficient, recoverable, and low-toxicity CO_2_-responsive green solvents.

## 1. Introduction

Growing global energy demand continues to drive the exploration and development of unconventional oil and gas resources. Although renewable energy is expanding rapidly, petroleum and natural gas are expected to remain major components of the global energy supply in the foreseeable future [1]. Consequently, the use of oil-based drilling fluids has increased substantially, leading to the generation of large quantities of oil-based drilling cuttings (OBDCs), one of the most important hazardous wastes produced by the oil and petrochemical industries [2,3,4]. OBDCs are complex multiphase mixtures containing petroleum hydrocarbons, polycyclic aromatic hydrocarbons, heavy metals, and various chemical additives [5,6]. Improper disposal can result in the release of OBDCs into soil, groundwater, and surrounding ecosystems through leaching, volatilization, and surface runoff, degrading soil quality, disrupting microbial communities, inhibiting plant growth, and promoting the accumulation of toxic substances throughout the food chain, posing long-term risks to both environmental and human health [7,8]. Owing to these hazards, OBDCs are classified as hazardous waste in many regulatory frameworks, and their safe treatment and resource recovery have become important challenges for the sustainable development of the oil and gas industry [5]. Current OBDC treatment technologies include reinjection, incineration, solidification, and solvent extraction [9]. Among these approaches, solvent extraction is widely regarded as a promising resource-recovery strategy because of its high oil recovery efficiency and rapid treatment efficiency [10]. However, conventional extraction solvents, such as dichloromethane, n-hexane, and other chlorinated hydrocarbons, are often associated with high toxicity, significant volatility, and energy-intensive recovery processes. These limitations hinder the sustainability of solvent-based treatment technologies [11,12,13]. Therefore, the development of green extraction solvents that combine high extraction efficiency, low toxicity, and efficient recoverability has become a critical step toward advancing the sustainable treatment and resource utilization of oil-based drilling cuttings.

CO_2_-switchable solvents (CSSs) have emerged as a promising class of responsive green solvents to overcome the trade-off between extraction efficiency and environmental sustainability associated with conventional extraction solvents. CO_2_-switchable solvent systems can be broadly distinguished according to the physicochemical property being reversibly altered. Switchable-polarity solvents (SPSs) undergo a substantial change in solvent polarity upon exposure to CO_2_, whereas switchable-hydrophilicity solvents (SHSs) reversibly switch between a relatively hydrophobic, water-immiscible or poorly miscible form and a hydrophilic, water-miscible form. Switchable-water (SW) systems, in contrast, rely on CO_2_-responsive additives to reversibly alter the physicochemical properties of an aqueous phase. The present study is primarily concerned with the molecular-design principles relevant to SHS-type systems, in which protonation of amine functionalities by CO_2_/H_2_O increases molecular hydrophilicity and subsequent CO_2_ removal restores the neutral, more hydrophobic form [14,15]. The CSSs mainly consist of tertiary amines or amidines as the main components, and CO_2_ was used as a reversible molecular switch to achieve efficient separation and recycling of the extractant by altering the hydrophilic and hydrophobic properties of the extraction system [16]. Under CO_2_-free conditions, CSSs remain hydrophobic and readily dissolve petroleum hydrocarbons [17]. Upon CO_2_ introduction, the amine groups react with CO_2_ and water to form hydrophilic ammonium bicarbonate species, resulting in a substantial increase in solvent polarity and facilitating phase separation. Subsequent CO_2_ removal by heating or inert-gas purging restores the original hydrophobic state, allowing solvent regeneration and reuse [13,18]. The effectiveness of this switching mechanism has been demonstrated in a variety of separation systems. For example, N,N-dimethyl-N-dodecylamine-based microemulsions undergo reversible transitions between single-phase and biphasic states under alternating CO_2_/N_2_ treatment [19]. Similarly, cyclohexyldimethylamine has achieved asphalt removal efficiencies exceeding 94% during oil-sand processing while maintaining low solvent loss [20]. These studies highlight the potential of CSSs to combine high extraction efficiency with efficient solvent recovery. Among the reported CSSs, N,N-dimethylcyclohexylamine has attracted particular attention because of its rapid CO_2_ responsiveness and excellent extraction capability [19]. A surfactant-free microemulsion system based on this solvent reduced the residual oil content of oil-based drilling cuttings to only 0.54%, demonstrating outstanding oil removal efficiency [21]. However, despite these functional advantages, N,N-dimethylcyclohexylamine has been associated with oral toxicity, skin irritation, and potential aquatic toxicity, raising concerns regarding its large-scale environmental application [22]. Consequently, the development of next-generation CSSs that maintain high switching efficiency and extraction efficiency while minimizing toxicity and environmental risks remains a big challenge.

Computer-aided molecular design (CAMD), a rational molecular engineering approach based on theoretical simulations, has demonstrated considerable potential in recent years across fields such as pharmaceuticals, chemical engineering, environmental science, and materials science [23]. Density functional theory (DFT) calculations performed using Gaussian 16 Revision A.03 WIN64 can construct an equivalent crude-oil solvent environment and calculate the standard Gibbs free energy change (ΔG) associated with the protonation reaction between solvent molecules and CO_2_ under dielectric and polarization conditions that closely resemble those of real systems. This provides a basis for analyzing the switching behavior of CSSs and characterizing their extraction efficiency [24]. Molecular dynamics (MD) simulations, using simulation platforms such as GROMACS, reveal the interaction between solvent molecules and environmental receptors such as biological enzymes at the atomic level, providing microscopic insights into the biodegradability of solvents and forming an important part of green chemistry design and evaluation [25]. In addition, machine learning (ML) algorithms can assist in identifying structure–performance relationships and screening candidate solvents, thereby supporting the rational optimization of CSSs [26]. However, although the CO_2_-responsive extraction and recovery characteristics of CSSs have been demonstrated, the molecular determinants governing their thermodynamic behavior and extraction performance remain insufficiently understood. In particular, the effects of molecular structure, modification position, and functional-group type, as well as their interactions, have not been systematically evaluated within a unified molecular-design framework. Moreover, extraction performance alone is insufficient to comprehensively evaluate CSSs, as solvent recovery characteristics and potential toxicity should also be considered during molecular optimization. Therefore, a systematic computational strategy integrating structural modification, thermodynamic evaluation, extraction performance, recovery characteristics, and toxicity assessment is needed for the rational development of CSSs.

In this study, CO_2_-switchable solvents (CSSs) are broadly defined as solvent systems whose polarity, hydrophilicity, or phase behavior can be reversibly altered through CO_2_-triggered chemical equilibria, particularly protonation/deprotonation of amine functionalities. Among these systems, switchable-hydrophilicity solvents (SHSs) represent an important subclass in which the solvent reversibly changes between a relatively hydrophobic state and a more hydrophilic state upon CO_2_ addition and removal. The present study focuses on the molecular-design principles relevant to SHS-type CO_2_ switching for petroleum extraction and solvent regeneration. A petroleum-like solvent environment was first constructed, and the Gibbs free energy changes in the CO_2_-switching reactions of the CSSs were calculated using Gaussian to characterize the structural dependence of their thermodynamic behavior. Based on these results, systematic molecular modifications were performed by regulating the hydrophobic tail structure and introducing hydroxyl, phenyl, ester, and combined functional groups at different positions within the hydrophobic linker region. Linear mixed-effects modeling was subsequently applied to quantify the effects and interactions of modification sites and functional-group types on extraction performance, while machine learning was further employed to identify key molecular descriptors associated with the thermodynamic response and elucidate the structure–performance relationships of CSSs. Finally, recovery calculations and toxicity prediction were incorporated to evaluate the recovery characteristics and predicted safety profiles of the optimized molecules. Through this integrated computational framework, this study aims to identify CSS candidates with enhanced extraction performance while maintaining favorable recovery characteristics and predicted safety profiles, thereby providing a structure-guided theoretical basis for the rational design and further experimental evaluation of CSSs.

## 2. Results and Discussion

### 2.1. Evaluation of the Extraction Efficiency of CSSs

In this study, the ${∆G}_{0}$ was used as a relative indicator of the propensity of CO_2_-switching extraction reactions to occur in CSSs. The calculations were performed using a pseudo-petroleum continuous-medium model. The resulting ${∆G}_{0}$ values were generally positive, which is consistent with the operational characteristics of CSSs, whose switching reactions typically require external stimuli under practical conditions. Therefore, the relative magnitude of ${∆G}_{0}$ can be used to compare the responsiveness of CSSs with different molecular structures and to evaluate potential to undergo CO_2_-triggered polarity transitions.

As shown by the molecular structures and computational results presented in Table 1, the ${∆G}_{0}$ values of linear tertiary amines (No. 1–7) and cyclic/aryl tertiary amines (No. 8–10) were primarily distributed within the range of 0.11–0.19 Hartree. The minimum ${∆G}_{0}$ value was approximately 0.1154 Hartree (No. 10), whereas the maximum value reached approximately 1.3403 Hartree (No. 13). As shown in Table 1, the calculated ΔG values for the linear tertiary amines (No. 1–7) were narrowly distributed between 0.164 and 0.168 Hartree, while the cyclic and aromatic amines exhibited a somewhat broader range. In contrast, the polyamine-containing compounds No. 13 and No. 14 showed substantially larger overall ΔG values. However, these values should be interpreted cautiously because the corresponding molecules contain multiple amine sites, whereas the monoamine compounds contain only one amine center. The calculated ΔG values therefore represent the overall thermodynamic responses of the modeled CO_2_-switching transformations and are not normalized on a per-amine or per-protonation basis. Consequently, the absolute values for the polyamine and monoamine systems should not be directly compared to infer intrinsic basicity or first-protonation energetics. In the subsequent structural-modification analysis, comparisons were therefore primarily made relative to the corresponding parent structure rather than between absolute ΔG values of chemically different amine classes.

### 2.2. Molecular Modification of CSSs

(1) Molecular structures of modified CO_2_-switchable solvents derived from No. 1–7

In this study, hydrophobic tail length was initially considered as the primary structural variable. Because No. 1–7 are all linear alkyl-substituted tertiary amines, the carbon-chain length of their hydrophobic tail regions increases systematically, providing a well-defined comparative framework. Therefore, no additional modifications were introduced to the hydrophobic tail region of these molecules.

Subsequently, modifications were performed in the hydrophobic linker region according to the strategy described in Section 3.2(2). No. 2–7 were selected as the parent molecules for structural optimization. While maintaining the central linker region unchanged, functional groups were introduced at the C2, C3, and C4 positions of the hydrophobic linker region, excluding structures that violated chemical valence requirements. The molecular structures of the designed CSSs are shown in Figure 1, with C3 position modifications presented as representative examples.

(2) Structural modifications of No. 8–15 CSSs

For No. 8–15 CSSs, the hydrophobic tail structure is inconsistent, or the original hydrophobic tail length deviates from the mainstream chain length. First, the modification strategy described in Section 3.2(1) is applied to normalize the hydrophobic tail region to the C_8_-equivalent hydrophobic tail, as shown in Figure 2a. Based on these C_8_-standardized structures, the modified No. 8–15 molecules were subsequently used as parent molecules for further structural optimization. Functional groups were introduced at the C2, C3, and C4 positions of the hydrophobic linker region while maintaining the central linker region unchanged. Structures that did not satisfy the modification criteria or violated chemical valence requirements were excluded. Representative molecular structures resulting from C3 position modification are shown in Figure 2. This series of modifications was designed to investigate whether the introduction of different functional groups into the hydrophobic linker region could enhance the extraction efficiency of CSSs after standardization of the hydrophobic tail to a C_8_ chain with favorable overall efficiency. In addition, these modifications provide a means to further regulate the hydrophilic–hydrophobic balance, electronic effects, and interaction capability with CO_2_, thereby enabling rational optimization of switching thermodynamics and extraction efficiency.

### 2.3. Analysis of the Extraction Efficiency of Novel CSSs

Based on the structural partitioning and systematic modification strategies applied to 15 CSSs, a total of 163 novel CSSs were designed in this study. By directly comparing the ΔG before and after structural modification, the effects of different modification strategies on the thermodynamic behavior of the CO_2_-switching extraction reaction were quantitatively evaluated. The corresponding changes in ΔG were further used to characterize the extraction efficiency change rate and to assess the effectiveness of each structural modification strategy. Under extraction conditions, the Gibbs free energy values of CO_2_, H_2_O, and HCO_3_^−^ are summarized in Table S1, while the Gibbs free energy values and associated calculation data for the extraction reactions of all newly designed CSSs are provided in Tables S2–S13. The extraction efficiency change rates of all modified CSSs are summarized in Table 2. According to the classification criteria adopted in this study, molecules with *φ* ≥ 50% were classified as highly effective modified molecules, whereas those with 50% ≥ *φ* ≥ 15% were classified as effectively modified molecules. Based on these criteria, 14 highly effective modified molecules and nine effectively modified molecules were identified, yielding a total of 23 modified CSSs with improved extraction efficiency.

#### 2.3.1. Analysis of the Extraction Efficiency Following Hydrophobic Tail Standardization

To eliminate the influence of the hydrophobic tail structure variation in CSSs 8–15, all molecules were first normalized to the C_8_-equivalent hydrophobic tail, and then the Gibbs free energy was calculated in a petroleum-like environment. This standardization minimized the influence of differences in hydrophobic tail size on the analysis of reaction free energy. As shown in Table 2, the extraction efficiency change rates of most modified molecules remained within ±10%, indicating that hydrophobic tail standardization has only limited effects on the Gibbs free energy of the extraction reaction. These results suggest that the hydrophobic tail region contributes little to the thermodynamic driving force of CO_2_ addition at the amine center. An exception was observed for tetramethylenediamine (No. 13), whose extraction reaction Gibbs free energy decreased markedly from 1.3403 to 0.2470 after C_8_-equivalent hydrophobic tail modification, corresponding to an extraction efficiency change rate of 81.57%. This result indicates that hydrophobic tail modification may have induced substantial changes in the reactant or product conformation, ion-pairing interactions, or solvation state during the CO_2_-switching reaction of tetramethylenediamine.

Overall, hydrophobic tail modification primarily alters the overall hydrophobicity and solvent environment of most CSSs, while exerting only a limited influence on the CO_2_/H_2_O reaction occurring at the amine center. Its effects are therefore more likely to be reflected in macroscopic properties, such as phase behavior, mass-transfer efficiency, and phase-separation efficiency [27]. Using this modification strategy, one highly effective modified CSS was identified, namely the C8-equivalent hydrophobic tail derivative of No. 13.

#### 2.3.2. Extraction Efficiency Analysis of Functional-Group Modifications in the Hydrophobic Linker Region

Based on the modification strategy, the 15 parent compounds were divided into two groups (i.e., No. 1–7 and No. 8–15). Group No. 1–7 constitute a control system with progressively increasing linear alkyl chain lengths and were used to characterize the effect of hydrophobic chain length on the thermodynamics of the extraction reaction. In contrast, group No. 8–15 was first standardized to a uniform C_8_-equivalent hydrophobic tail to establish a consistent hydrophobic background, thereby minimizing the influence of differences in hydrophobic scale and enabling the effects of molecular structure on extraction efficiency to be evaluated independently. On this basis, four modification strategies—hydroxyl-group, phenyl-group, ester-group, and combined hydroxyl- and phenyl-group modifications—were introduced into the hydrophobic linker region at the C2, C3, and C4 positions of all molecules. The extraction efficiency change rate (*φ*) was used as the evaluation metric (Table 2). The effects of modification site and functional-group type were systematically analyzed using a linear mixed-effects model, in which the parent molecule (No. 1–15) was treated as a random effect (Figure 3), while modification site and functional-group type were treated as fixed effects. In addition, Table 3 presents the mean effect matrix for each modification site–functional group combination.

Overall, the modification responses differed markedly between groups No. 1–7 and No. 8–15. For molecules in group No. 1–7, most functional-group modifications produced negative effects, with extraction efficiency change rates (*φ*) primarily ranging from −5% to −20%. In particular, phenyl-group and ester-group modifications consistently exhibited inhibitory effects, typically within the range of −14% to −17%. Notably, modifications at the C3 position produced exceptionally large negative responses, with φ values of −300.86% for No. 5 and −306.89% for No. 7. These results suggest that structural perturbations at the C3 position may substantially disrupt the reaction configuration or the CO_2_-switching process. In contrast, molecules in group No. 8–15 exhibited a much broader distribution of *φ* values. In addition to the generally positive effects observed for several molecules (e.g., No. 13, which exhibited *φ* values exceeding 80% at multiple modification sites), both exceptionally strong enhancement (No. 14, C4 position phenyl-group modification, *φ* = 278.43%) and pronounced inhibition (No. 12, C4 position combined hydroxyl- and phenyl-group modification, *φ* = −861.10%) were observed.

As summarized in Table 3, several site-functional group combinations in the C8-standardized system exhibited positive mean effects, including C4-OH (0.247) and C4-ph (0.1604). These results indicate that, after standardization of the hydrophobic background, the influence of functional-group modification shifts from being largely suppressed to becoming the dominant factor governing extraction efficiency. This trend is consistent with the chain length–functional group coupling mechanism previously reported for CSSs [15]. Furthermore, the LMM indicates that the differences between the two molecular systems arise primarily from significant interactions among the hydrophobic tail, modification site, and functional-group type, rather than from the additive effects of individual factors alone.

(1) Analysis of the extraction efficiency based on modification sites

The influence of modification sites on extraction efficiency exhibited pronounced spatial dependence. The C2 position, located adjacent to the amine center, showed relatively small variations in extraction efficiency. For molecules No. 1–7, hydroxyl-group modification produced extraction *φ* that were generally within ±2%. In the C_8_-standardized system, although positive effects were observed for several molecules, most *φ* values remained below 15%, indicating that the C2 position is still dominated by the local electronic environment of the amine center, thereby limiting the influence of structural modification.

In contrast, the C3 position exhibited the highest structural sensitivity. In addition to the exceptionally large negative φ values observed in molecules No. 1–7 (exceeding −300%), substantial fluctuations were also observed in the C8-standardized system. As summarized in Table 3, the mean effects of phenyl-group and ester-group modifications at the C3 position were −0.4177 and −0.1900, respectively, which were markedly lower than those at the other modification sites. These results indicate that the C3 position is highly sensitive to structural perturbations. As suggested by density functional theory studies, such spatial effects may enhance entropic contributions to the reaction process [28]. The C4 position, by comparison, exhibited a transition from predominantly negative effects to more differentiated responses. In molecules No. 1–7, φ values were generally distributed between −10% and −15%, whereas in the C8-standardized system, several molecules (e.g., No. 13 and No. 14) showed pronounced positive effects. This finding suggests that modifications at the C4 position can alter the reaction behavior by influencing the overall molecular conformation or intermolecular interactions.

Mechanistically, the site-dependent effects reflect a transition from local electronic control to global structural regulation. Specifically, the C2 position is primarily governed by local electronic and steric interactions around the amine center, the C3 position represents a highly sensitive region where electronic and conformational effects are strongly coupled, and the C4 position mainly influences the reaction through changes in molecular conformation and solvation. The LMM further indicates that the main effect of modification site alone is relatively limited, whereas its influence depends strongly on the type of functional-group modification. In particular, the interaction between modification site and functional-group type is most pronounced at the C3 position, accounting for the extreme *φ* values observed in this study.

(2) Analysis of the Effects of Different Functional-Group Modifications

The effects of different functional-group modifications differed substantially between groups No. 1–7 and No. 8–15. In molecules of group No. 1–7, hydroxyl-group modification exerted only a limited influence, with φ generally remaining close to zero. In contrast, hydroxyl-group modification significantly enhanced extraction efficiency in the C8-standardized system. For example, molecule No. 13 exhibited a φ value of 86.91% following C2 position hydroxyl-group modification. Consistent with these observations, Table 3 shows that hydroxyl-group modification produced positive mean effects at all modification sites (0.0506–0.2497), indicating that enhanced polarity and hydrogen-bonding interactions are more effectively expressed in a uniform hydrophobic environment. This finding is consistent with previous studies demonstrating the role of polar functional groups in regulating the response behavior of CSSs [15].

Phenyl-group modification generally exhibited inhibitory effects in molecules from group No. 1–7 but became favorable for several site–molecule combinations in the C8-standardized system. For example, the mean effect of C4 position phenyl-group modification reached 0.1604, and several molecules exhibited pronounced improvements in extraction efficiency. These results suggest that the π-conjugated aromatic structure can enhance intermolecular interactions when combined with an appropriate molecular conformation and hydrophobic environment [29]. In contrast, ester-group modification consistently produced negative effects, with a mean effect of −0.1900 at the C3 position (Table 3), indicating that the combined influence of electron-withdrawing effects and steric constraints suppresses the extraction reaction. Combined hydroxyl- and phenyl-group modification exhibited the greatest variability, with mean effects alternating between positive and negative depending on the modification site (e.g., 0.0560 at C3 and −0.4568 at C4), reflecting the complex synergistic and antagonistic interactions between multiple functional groups.

Overall, the effects of functional-group modification are strongly structure-dependent rather than intrinsically favorable or unfavorable. The same functional group can therefore produce markedly different, or even opposite, regulatory effects depending on the modification site and hydrophobic background. The linear mixed-effects model further indicates that, although the main effect of functional-group type is statistically significant, its contribution is smaller than that of its interactions with modification site and hydrophobic environment. In particular, the higher-order interaction terms associated with combined modifications account for the largest fluctuations in extraction efficiency.

Comparison of molecules from groups No. 1–7 and No. 8–15 further demonstrates the coupling between hydrophobic background, modification site, and functional-group type. For molecules from No. 1–7, differences in hydrophobic chain length exert a masking effect on functional-group modifications, resulting in predominantly negative responses. After hydrophobic tail standardization in molecules No. 8–15, this masking effect is largely eliminated, allowing the synergistic effects between modification site and functional-group type to become fully apparent and producing substantial efficiency enhancement in specific molecular structures. Overall, the sensitivity of extraction efficiency to modification site follows the order C3 > C4 > C2, consistent with the pronounced negative mean effect observed for the C3 position in Table 3. The LMM further indicates that changes in extraction efficiency are governed not by a single structural factor but by the multilevel coupling among modification site, functional-group type, and hydrophobic environment. Among these factors, the interaction between modification site and functional-group type is the primary determinant of extraction efficiency, whereas the hydrophobic tail acts as a modulating factor controlling the magnitude of these interactions. Higher-order interaction effects explain the extreme enhancement and inhibition observed for molecules such as No. 14 and No. 12. Collectively, these findings indicate that structural modification of the hydrophobic linker region is fundamentally a multifactorial regulatory process in which interaction effects play a dominant role in determining the overall response of the system.

### 2.4. Analysis of the Recovery Efficiency of Novel CSSs

To further evaluate the physicochemical feasibility of the 23 thermodynamically selected candidates, their neutral-state logKow values were estimated using EPI Suite [30]. The predicted values ranged from 1.33 to 5.88, with a mean value of 3.18, demonstrating substantial variation in hydrophobicity among the designed structures. Previous experimental studies have suggested that conventional monoamine SHSs generally exhibit logKow values of approximately 1.2–2.5, whereas diamine SHSs can tolerate a broader hydrophobicity range of approximately 2–5 [15,31]. The present results therefore indicate that not all thermodynamically favorable structures identified by the DFT screening are expected to function as conventional SHSs. In particular, several monoamine-derived candidates exhibit logKow values above the conventional SHS range, indicating a potential limitation in CO_2_-triggered transfer into the aqueous phase. In contrast, six No. 13 diamine derivatives, including 2-13-C-Ph, 3-13-C-Ph, 3-13-C-OH-Ph, 13-C, 4-13-C-Ph, and 4-13-C-OH-Ph, have predicted logKow values within the reported range for diamine SHSs and therefore represent higher-priority candidates for subsequent experimental phase-behavior evaluation.

A comprehensive evaluation of CSSs should consider both extraction and recovery efficiency. Although the analysis of extraction efficiency change rates identified several highly effective and effective modified molecules from a thermodynamic perspective, their practical applicability would remain limited if improvements in extraction efficiency were accompanied by a substantial deterioration in solvent recovery [32]. Therefore, the 23 highly effective and effective modified molecules (hereafter referred to as novel CSSs) identified in this study were further evaluated by calculating the ΔG of their recovery reactions and the corresponding recovery efficiency change rates in both ethanol and water recovery environments (Table 4). The Gibbs free energy values of CO_2_, H_2_O, and HCO_3_^−^, the selected novel CSSs, and their corresponding parent molecules are provided in Tables S14 and S15. To establish a unified evaluation criterion, the recovery efficiency change rate (φ) was classified into three categories: φ ≥ −5%, indicating a negligible effect on the recovery process; −15% ≤ φ < −5%, indicating an acceptable level of disturbance; and φ < −15%, indicating an adverse effect on solvent recovery.

In the ethanol recovery environment, the recovery efficiency change rates of most modified molecules were negative, ranging from −19.24% to +2.11%, with the majority falling between −5% and 0%. These results indicate that structural modification generally exerts only a slight adverse effect on the recovery process. A similar trend was observed in the water recovery environment, where the recovery efficiency change rates ranged from −20.32% to +3.46%. However, compared with ethanol, a greater number of CSSs exhibited positive recovery efficiency change rates in water, suggesting that water recovery is more favorable for preserving or enhancing solvent recyclability. Notably, several novel CSSs demonstrated the potential to simultaneously improve both extraction and recovery efficiency, indicating the possibility of achieving dual-stage enhancement. Among all modified molecules, 2-14-C-OH-Ph exhibited the poorest recovery efficiency in both recovery environments, with a minimum recovery efficiency change rate of −19.24%, which falls below the acceptance threshold of −15%. This molecule can therefore be excluded from further evaluation.

Combined evaluation of recovery efficiency in ethanol and water recovery environments (Table 4) showed that 69.6% of the novel CSSs satisfied the criterion of *φ* ≥ −5% in both solvents. 2-13-C-Ph, 3-13-C-Ph, 3-13-C-OH-Ph, 3-3-OH-Ph, 3-4-OH-Ph, 3-15-C-Ph, and 4-14-C maintained the enhanced extraction efficiency while causing a negligible reduction in recovery efficiency, and can therefore be regarded as preferred molecular structures. In addition, 17.4% of the novel CSSs exhibited recovery efficiency change rates within the acceptable range (−15% ≤ *φ* < −5%), indicating that functional-group modification within the hydrophobic linker region generally does not substantially impair the recovery capability of the CO_2_-switchable solvent system. Overall, the recovery-efficiency screening demonstrated that 87% of the novel CSSs identified in this study achieved the dual objective of enhanced extraction efficiency and stable recovery, confirming the effectiveness of the proposed molecular design strategy.

### 2.5. Analysis of the Thermodynamic Mechanisms Governing Reactions of Novel CSSs

To further elucidate how molecular structure regulates the thermodynamic behavior of CO_2_-switching reactions, a systematic analysis was conducted using the SVR-PCA model. By integrating model efficiency evaluation, residual analysis, and interpretation of key structural feature importance, the relationships between molecular structural parameters, CO_2_ responsiveness, and reaction Gibbs free energy were examined. This analysis provides further insight into the structural factors governing the thermodynamic behavior of CSSs.

#### 2.5.1. Efficiency Evaluation of the Thermodynamic Model for CSSs

Based on the preprocessed molecular descriptor dataset, an SVR-PCA model was constructed to predict the reaction Gibbs free energy (ΔG) of novel CSSs. To evaluate its predictive efficiency, the optimized model (C = 10, ε = 0.1, γ = 0.01, RBF kernel) was used to fit and predict all samples in the training set. The predicted values were subsequently transformed back to the original ΔG scale using the inverse Box–Cox transformation. The final model achieved a coefficient of determination (R2) of 0.9732, a root mean square error (RMSE) of 0.0122, and a mean absolute percentage error (MAPE) of 2.72%. These results demonstrate that the model exhibits excellent predictive efficiency, high accuracy, and strong reliability for characterizing the thermodynamic behavior of CO_2_-switching reactions [33,34,35].

The scatter plot of the predicted versus actual values (Figure 4a) shows that the data points are closely distributed around the y = x diagonal, with no obvious systematic deviation, indicating excellent agreement between the predicted and observed ΔG values for the training set. Residual analysis further showed a mean residual of −0.0002 (approximately zero), a standard deviation of 0.0122, and a maximum absolute residual of 0.0530, corresponding to sample 3-15-C-Ph. As shown in Figure 4b, the residuals are randomly distributed across the range of predicted values, with no apparent funnel-shaped or systematic pattern, satisfying the assumption of homoscedasticity [36]. These results indicate that the SVR-PCA model effectively captures the nonlinear relationship between molecular descriptors and ΔG, demonstrating high fitting accuracy, statistical reliability, and predictive stability.

#### 2.5.2. Analysis of Key Factors Influencing the Thermodynamic Effects of CSSs

To identify the key molecular structural features governing the ΔG of the CO_2_-switching reaction, the mutual information method was first used to select the 15 molecular descriptors most strongly associated with ΔG from the original feature set. A nonlinear SVR-PCA model was then constructed using these selected descriptors. The contribution of each principal component to model efficiency was subsequently evaluated using permutation importance analysis (Figure 5). Finally, the relationships between the key original molecular descriptors and ΔG were further examined through scatter plot analysis to elucidate the underlying thermodynamic mechanisms (Figure 6).

Fifteen molecular descriptors (i.e., ATSC5v, ATSC3p, ATSC1i, ATSC3i, ATSC4i, ATSC5i, SpMin1_Bhp, VC-3, VPC-5, ETA_dBetaP, ETA_Eta_F_L, nAtomP, MLFER_BO, MLFER_S, and MLFER_E) were identified most strongly associated with ΔG by mutual information method. These descriptors primarily belong to the categories of centered Moreau–Broto autocorrelation descriptors (ATSC series), molecular connectivity indices (VC-3 and VPC-5), Extended Topochemical Atom (ETA) descriptors, molecular polarity-related descriptors (MLFER series), and atomic property descriptors (nAtomP) [37]. Notably, four descriptors from the ATSC series (i.e., ATSC1i, ATSC3i, ATSC4i, and ATSC5i) were selected simultaneously, indicating that the electrotopological states of specific atoms play an important role in determining the energy barrier of the CO_2_-switching reaction [38].

According to the permutation importance analysis (Figure 5), the first four principal components (i.e., PC1–PC4) contributed most substantially to the model efficiency, with PC3 exhibiting the highest importance. This result indicates that the third principal component, derived from the PCA transformation of the polynomial interaction features, contains the most influential information for predicting ΔG. Because each principal component represents a linear combination of multiple original molecular descriptors, its importance indirectly reflects the combined effects of these descriptors. The high contributions of multiple principal components further suggest that the thermodynamic behavior of the CO_2_-switching reaction is governed by the synergistic effects of multiple molecular characteristics, including electronic properties, steric effects, and hydrophobic chain structures, rather than by any single structural factor.

To further examine the relationships between the key molecular descriptors and ΔG, scatter plots were generated for the four most influential descriptors (i.e., ATSC5v, ATSC3p, ATSC1i, and ATSC3i) against ΔG (Figure 6). The trend lines in Figure 6 are provided as visual guides to illustrate the overall variation trends between the selected molecular descriptors and ΔG and are not intended for independent model fitting or prediction. Trend lines were fitted using the Savitzky–Golay smoothing method to better visualize the underlying response patterns. All four descriptors exhibited clear nonlinear relationships with ΔG, indicating that the thermodynamic behavior of the CO_2_-switching reaction is not governed by a simple linear dependence on any single molecular descriptor. Instead, it is regulated by the combined effects of multiple molecular characteristics, including electronic properties and steric effects.

Among the selected descriptors, ATSC5v is a centered Moreau–Broto autocorrelation descriptor weighted by van der Waals volume, reflecting the distribution of molecular spatial volume over topological distances [39,40]. As shown in Figure 6a, ΔG varies only slightly over the low-to-moderate range of ATSC5v and exhibits a slight decreasing trend in some regions. However, ΔG increases markedly as ATSC5v continues to increase. This trend suggests that increasing molecular spatial volume and hydrophobic chain length may enhance steric hindrance and conformational constraints, thereby reducing the thermodynamic stability of the CO_2_ addition process [41]. In particular, the rapid increase in ΔG at high ATSC5v values indicates that excessive molecular volume may hinder the approach of CO_2_ to the tertiary amine reaction site and weaken the stability of the reaction intermediate; ATSC3p is a polarizability-weighted centered Moreau–Broto autocorrelation descriptor that reflects the molecular electron cloud polarizability [40,42]. As shown in Figure 6b, ΔG increases sharply with increasing ATSC3p in the lower descriptor range, reaches a local maximum near −5, and then decreases rapidly before gradually stabilizing. This pattern indicates a threshold effect of molecular polarizability on the CO_2_-switching reaction. Within a specific range, increasing polarizability may destabilize the electronic distribution of the system, resulting in higher reaction free energy. Beyond this threshold, however, greater polarizability appears to stabilize the polar structure and ionized intermediates formed after CO_2_ addition, thereby improving the thermodynamic stability of the system [43]; ATSC1i is an ionization potential-weighted centered Moreau–Broto autocorrelation descriptor that primarily reflects the local electron-donating ability and electronic distribution of the molecule [44]. As shown in Figure 6c, ΔG remains relatively high at low ATSC1i values but decreases substantially as ATSC1i increases before approaching a stable plateau. This trend suggests that enhancing the electron-donating ability around the tertiary amine center promotes its nucleophilic reactivity toward CO_2_, thereby facilitating the CO_2_ addition reaction and reducing the ΔG of the system. At higher ATSC1i values, the ΔG curve gradually levels off, indicating that once the electronic effect reaches a certain level, its contribution to improving thermodynamic stability becomes progressively less pronounced; ATSC3i is also an ionization potential-weighted centered Moreau–Broto autocorrelation descriptor but characterizes the electronic distribution over longer topological distances [45]. As shown in Figure 6d, ΔG initially decreases with increasing ATSC3i, exhibits pronounced fluctuations with local maxima in the intermediate range, and then gradually stabilizes at higher values. This behavior suggests that a moderate degree of electron delocalization favors stabilization of the reaction intermediate. However, excessive complexity in the electronic distribution or enhanced intramolecular electron redistribution may induce local electronic competition and conformational changes, leading to substantial fluctuations in ΔG [46].

By analyzing the variation patterns of the four key molecular descriptors, it can be inferred that the thermodynamic behavior of CO_2_-switchable solvent reactions is governed by the combined effects of electronic properties, steric effects, and molecular conformational flexibility. Enhanced electron-donating ability generally promotes the nucleophilic addition of CO_2_, whereas moderate molecular polarizability contributes to the stabilization of polar intermediates. In contrast, excessive molecular volume or overly complex electronic distributions may increase steric hindrance and reduce the thermodynamic stability of the reaction. Overall, solvent molecules with moderate alkyl chain lengths, balanced molecular branching, and appropriate polar functional groups tend to exhibit more favorable ΔG values, suggesting a better balance between CO_2_ responsiveness and thermodynamic stability. These findings provide useful guidance for the molecular design of next-generation CSSs. In particular, optimizing the electronic environment around the amine center, tailoring hydrophobic chain length, and introducing suitable polar functional groups may offer an effective strategy for improving the thermodynamic efficiency of CO_2_-switching reactions.

### 2.6. Prediction and Evaluation of the Toxicity of Novel CSSs Using ADMETlab 3.0

To evaluate the environmental and human health safety of the designed CSSs, this study performed a comprehensive toxicity assessment using the ADMETlab 3.0 platform. A total of 24 toxicity endpoints were predicted for 23 selected novel CSSs and their corresponding parent molecules. The summarized prediction results are presented in Table S15 [47]. Detailed prediction results for all 24 toxicity endpoints for both the parent and modified CSSs are provided in Table S16, and a comparative heatmap of the toxicity profiles is shown in Figure 7.

Based on the prediction of 24 toxicity endpoints for 23 selected novel CSSs and their corresponding parent molecules using the ADMETlab 3.0 platform, structural modification was found to influence the toxicity profiles of the designed solvents. Overall, structural modifications altered the distribution of toxicity endpoints, although the effects varied depending on the modification site and functional group. In particular, the introduction of hydroxyl (-OH) and ester (-COO) groups generally reduced several key toxicity endpoints, including drug-induced liver injury (DILI), AMES toxicity, carcinogenicity, and drug-induced nephrotoxicity (DI-Neuro). For most modified molecules, the predicted values for these endpoints were lower than those of their corresponding parent molecules. Among the modified structures, 4-14-COO showed improved efficiency in 16 of the 24 evaluated toxicity endpoints relative to its parent molecule, with consistently lower predicted toxicity for hepatotoxicity, nephrotoxicity, respiratory toxicity, and several ecotoxicity endpoints. These results suggest that introducing an ester group into the No. 14 parent structure may reduce multiple systemic toxicity risks.

In contrast, benzene ring (-Ph) modification improved some ecotoxicity and cytotoxicity endpoints in certain molecules, but its effects were less consistent and depended on both the modification site and the parent structure. Moreover, ecotoxicity endpoints did not exhibit a uniform decreasing trend across all modified molecules, indicating that structural modification influences environmental toxicity in a complex manner and may involve trade-offs among different toxicity endpoints [48]. Therefore, evaluating molecular safety based on a single toxicity endpoint is insufficient, and a comprehensive assessment using multiple toxicity endpoints is required.

Further analysis of different toxicity categories indicates that organ toxicity endpoints are particularly sensitive to structural modification. In particular, DI-Neph and DI-Neuro decreased for most modified molecules, which may be associated with changes in molecular polarity that alter in vivo distribution and reduce accumulation in specific organs [49]. Environmental toxicity endpoints also exhibited an overall improvement. The bioconcentration factor (BCF) generally decreased, whereas the aquatic toxicity parameters LC50DM and LC50FM generally increased, indicating reduced predicted aquatic toxicity. These results suggest that the modification strategies decreased molecular hydrophobicity and persistence, thereby lowering potential ecological risks. This finding is particularly relevant because CSSs are frequently applied in processes involving aqueous environments. In addition, several local exposure-related toxicity endpoints, including skin sensitization, eye corrosion, eye irritation, and respiratory toxicity, responded to structural modification. Although skin sensitization changed only slightly for most modified molecules, reductions in eye corrosion, eye irritation, and respiratory toxicity were observed for several compounds, with 4-14-COO and 2-12-Ph showing the greatest improvements. These results suggest that increasing molecular polarity may weaken hydrophobic interactions with biological membranes, thereby reducing the potential for local tissue irritation [50].

Based on the overall distribution of toxicity endpoints shown in Figure 7, several structurally optimized molecules with favorable toxicity profiles can be identified. Among them, 4-14-COO, 4-14-OH, 4-13-OH, 3-13-OH, 3-11-OH-Ph, 2-14-OH-Ph, 2-13-OH, and 2-12-Ph exhibited reduced predicted toxicity at multiple endpoints, with particularly consistent improvements in hepatotoxicity. It should be noted that none of the 23 selected novel CSSs simultaneously showed reduced predicted toxicity across all 24 evaluated endpoints. Therefore, the preferred solvent should be selected according to the specific requirements of the intended application. Overall, these results demonstrate that rational molecular structural modification can improve the functional efficiency of CSSs while reducing predicted toxicity across multiple endpoints, providing valuable structural guidance and a theoretical basis for the rational design of next-generation green solvents.

## 3. Materials and Methods

### 3.1. Screening of CO_2_-Switchable Solvent Molecules

To investigate the influence of molecular structure on the CO_2_-responsive behavior of CSSs, N,N-dimethylcyclohexylamine, a widely used CSS known for its high responsiveness and excellent degreasing efficiency, was used as the core reference structure [14,21,51,52,53]. A total of 15 common tertiary amine-based solvents were selected as representative compounds, covering a broad range of structural types, including linear alkylamines, cycloalkylamines, aromatic amines, polyamines, and amines containing ether or hydroxyl functional groups. This selection was intended to systematically evaluate the effects of key structural features, such as hydrophobic tail length, ring structures, and hydrophilic functional groups, on solvent properties (Table 5). The 15 parent compounds used in this study were not all assumed to be experimentally established SHSs. Instead, they were selected as a structurally diverse amine-based reference set, including reported SHSs, CO_2_-responsive amines, and related switchable amphiphilic structures, to systematically examine how molecular architecture affects CO_2_-triggered protonation thermodynamics and to guide the subsequent design of CSS candidates.

### 3.2. Modification Strategies for CO_2_-Switchable Solvent Molecules

The reversible response behavior of CSSs originates from the distinct chemical functions of different structural segments before and after exposure to CO_2_ [14,64,65]. Based on their molecular structures, the 15 parent compounds were divided into four functional regions: the hydrophobic tail region, the central linker region, the hydrophobic linker region, and the heteroaromatic region.

First, the hydrophobic tail region was defined as the structural unit directly connected to the amine group that primarily contributes to molecular hydrophobicity and nonpolar character. This region is typically composed of linear alkyl chains, cycloalkyl groups, or aromatic substituents and plays a key role in determining the solubility, partitioning behavior, and phase-separation characteristics of CSSs in oil-phase environments [64]. Among the 15 selected CSSs, the hydrophobic tail region exhibits systematic structural variation, ranging from short-chain and long-chain alkyl groups to cycloalkyl and aromatic substituents. This diversity provides a structural basis for subsequent analyses of the relationship between hydrophobic volume and switching behavior.

Second, the carbon- or heteroatom-based framework that is not directly attached to the amine nitrogen but forms the core backbone of the molecule is defined as the central linker region. This region corresponds to the α-carbon or α-atom linkage adjacent to the amine nitrogen and serves as the primary structural domain in which electronic changes occur during CO_2_-induced reactions, such as addition, proton transfer, and ion-pair formation [66]. The rigidity, length, and bonding patterns of the central linker region vary among different molecules, and these structural differences directly affect the thermodynamic stability of the CO_2_-binding process during amine reactions [67].

Furthermore, the carbon chain or substituent groups surrounding the amine center, but not directly involved in the reaction, are defined as the hydrophobic linker region. This region consists of alkyl substituents attached to the nitrogen atom or carbon-chain structures connected to the amine at adjacent positions. Its primary role is to regulate the local hydrophobic environment and electronic effects around the amine center, thereby indirectly influencing the reversibility and stability of the CO_2_-responsive process [68]. In polyamine and branched-amine systems, the hydrophobic linker region represents a key structural domain that contributes to molecular complexity and influences variations in CO_2_-responsive behavior [69].

In addition, the aromatic or heteroaromatic ring structures present in certain molecules are defined as a distinct heteroaromatic region. This region consists primarily of aromatic and heteroaromatic systems and, although not universally present among all molecules, can substantially influence solvent polarizability, π–π interaction characteristics, and compatibility with the oil phase [67]. Therefore, the heteroaromatic region serves as a selectable structural domain for investigating the effects of aromaticity on the overall performance of CSSs.

Through the four-segment structural framework described above, the 15 CSSs, despite exhibiting substantial variation in their functional groups and substituents, can be systematically compared within a unified structural classification scheme. This framework not only facilitates the identification of the roles of individual structural regions in CO_2_-responsive behavior but also provides a structural basis for subsequent free energy analyses and molecular optimization in crude oil-equivalent solvent environments. Detailed structural segmentation information is presented in Figure 8.

Based on the four functional regions, fifteen CSSs were modified and evaluated for the CO_2_-responsive behavior in petroleum-like environments following the two steps below.

(1) Modification of the hydrophobic tail to a C8-equivalent structure

The hydrophobic tail regions of CSSs (e.g., N,N-dimethylcyclohexylamine, N,N-dimethylaniline, and triethylamine), where the hydrophobic tail structures are inconsistent, were replaced by a C_8_-equivalent structure. Previous studies have shown that medium-chain hydrophobic tails, represented by C_8_, exhibit superior efficiency in regulating CO_2_-related interfacial and phase behavior. Previous studies have shown that medium-chain hydrophobic tails, represented by C8, can provide a suitable balance among oil-phase compatibility, hydrophobic–hydrophilic characteristics, phase-partitioning behavior, and molecular mobility [15,28]. Thus, the original hydrophobic tail length deviates from the mainstream chain length to C_8_-equivalent hydrophobic tails, thus eliminating interference caused by differences in hydrophobic tail size.

(2) Introduction of functional groups into the hydrophobic linker region of CSSs

The introduction of hydroxyl groups into the hydrophobic linker region of CSSs can enhance the local polarity and hydrogen-bonding capacity around the amine center [14,15]. To avoid introducing chemically labile α-hydroxyamine motifs, the carbon atom directly bonded to the amine nitrogen was not selected for hydroxyl functionalization. In the numbering scheme adopted in this study (Figure 9), this directly bonded carbon is defined as C1, whereas C2, C3, and C4 represent progressively more distal positions along the hydrophobic linker. Accordingly, hydroxyl and phenyl groups were introduced only at C2–C4, while ester groups were introduced at C3 and C4 [15,70,71,72]. Thus, none of the designed hydroxyl-modified structures contains an N–C(OH) hemiaminal motif [15]. These modification positions were selected to systematically evaluate the effects of functional-group type and position on molecular polarity and CO_2_-triggered switching thermodynamics while avoiding direct alteration of the amine center.

In addition, the central linker region serves as the structural framework connecting the amine center with the hydrophobic tail and other functional groups. This region directly influences molecular conformational stability and the spatial orientation of the amine group. To avoid introducing new reaction pathways or altering the fundamental molecular characteristics of the solvents, no modifications were made to the central linker region in any of the molecules examined in this study. Instead, this region was retained as a common structural framework for comparison among different CSSs. Among the 15 CSSs, only N,N-dimethylaniline and N,N-dimethylbenzylamine contain aromatic systems, whereas the remaining molecules do not possess heteroaromatic regions. These two compounds were used to evaluate the influence of aromatic π-systems on the free energy of CO_2_-switching reactions, and no further modifications were introduced into this region.

To facilitate the identification of the designed molecules, molecular labels follow the format “modification site–parent molecule number–modification strategy”. For example, “2-1-OH” denotes hydroxyl modification at the C2 position of parent molecule No. 1. For compounds whose hydrophobic tails were first standardized to a C8-equivalent structure, the suffix “C” is additionally included; for example, “4-14-C-COO” represents parent molecule No. 14 after C8-equivalent hydrophobic tail standardization and subsequent ester-group modification at the C4 position. The abbreviations C, OH, Ph, and COO denote C8-equivalent hydrophobic tail standardization, hydroxyl, phenyl, and ester modifications, respectively.

### 3.3. Characterization of Extraction and Recovery Efficiency in CO_2_-Switching Reactions—Gibbs Free Energy Calculations

Gaussian is a quantum chemistry software package capable of calculating molecular energies, reaction pathways, and spectroscopic properties based on quantum mechanical principles. In this study, Gaussian was employed to calculate the Gibbs free energy of CO_2_-switching reactions for CSSs under dielectric and polarization conditions that closely approximate those of real systems [24]. The calculated standard ΔG is a key thermodynamic parameter for evaluating the relative spontaneity of the reaction between CO_2_ and CSS molecules. It reflects the potential of CSSs with different molecular structures to undergo CO_2_-triggered polarity transitions and therefore provides a thermodynamic basis for analyzing their CO_2_-responsive behavior [73].

The general equation describing the CO_2_-switching reaction is as follows:

(1) $$R_{3}N+{\mathrm{CO}}_{2}+H_{2}O⇌[R_{3}\mathrm{NH}{]}^{+}+[{\mathrm{HCO}}_{3}{]}^{-}$$

#### 3.3.1. Thermodynamic Evaluation of CO_2_-Triggered Extraction Behavior of CSSs

##### Establishment of a Petroleum-like Environment for CSSs Extraction

As functional solvents with reversible CO_2_ responsiveness, CSSs typically operate in complex oil-phase or petroleum-like environments. To accurately evaluate the stability of CSS molecules and the associated changes in reaction free energy, a representative light Arabian crude oil was selected as the reference system for characterizing the operating environment of CSSs. Based on the SARA (Saturates, Aromatics, Resins, and Asphaltenes) classification system widely used in the petroleum industry, crude oil was treated as a continuous medium composed of multiple components [74]. The relative proportions of saturates (59.9%), aromatics (35.8%), polar components (1.54%), and asphaltenes (2.77%) were determined for the system [75]. Representative compounds were then selected for each component category: n-dodecane was used to characterize the nonpolar environment of the saturated hydrocarbon phase, toluene was used to represent the aromatic microenvironment associated with aromatic hydrocarbons and asphaltenes, and pyridine was used to characterize the weak polarity and hydrogen-bond-accepting properties of the polar fraction [74,76,77]. The complete solvent parameters for these representative compounds are available in the Minnesota Solvent Descriptor Database (Table 6). Finally, a weighted-average approach was employed to integrate key solvent parameters of n-dodecane, toluene, and pyridine, including the dielectric constant, optical constant, hydrogen-bond acidity/basicity, interfacial tension parameters, aromatic carbon ratio, and halogen electronegativity. This procedure enabled the construction of a solvent environment equivalent to crude oil. The weighted-average equation and calculation procedure (using relative permittivity as an example) are presented below.

(2) $$\varepsilon_{mix}=\sum_{i}w_{i}\varepsilon_{i}$$

(3) $$\varepsilon_{mix}=w_{dod}(2.0060)+w_{tol}(2.3741)+w_{pyr}(12.978)$$

In which Equation (2) represents the weighted-average method used to calculate the solvent parameter, $\varepsilon_{mix}$, of the equivalent crude oil environment through a linear weighting procedure. Here, $\varepsilon_{mix}$ denotes the weighted solvent parameter, $w_{i}$ represents the relative proportion of the i-th component in the crude oil system, and $\varepsilon_{i}$ is the experimental or empirical parameter value of the representative solvent for the i-th component obtained from the Minnesota Solvent Descriptor Database. Equation (3) illustrates the weighted-average calculation using the static dielectric constant (Eps) as an example. In this equation, $w_{dod}$, $w_{tol}$, and $w_{pyr}$ represent the relative proportions of n-dodecane, toluene, and pyridine, respectively, in the equivalent crude oil solvent environment.

##### Evaluation of the Extraction Efficiency of CSSs—Protonation Process

The influence of molecular structure on CO_2_-triggered switching was preliminarily evaluated by comparing the Gibbs free energy changes associated with amine protonation and bicarbonate formation. The change in ΔG before and after structural modification was used as a relative thermodynamic descriptor for evaluating how structural modification influences the propensity for CO_2_-triggered hydrophilic conversion. The change in ΔG before and after structural modification was used to characterize the rate of extraction efficiency (φ). As φ is a relative computational metric derived from DFT-calculated Gibbs free energy changes, it was used primarily for comparative evaluation of structural modifications rather than as a direct surrogate for experimentally measured extraction efficiency. For consistency in numerical reporting, φ values were uniformly rounded to two decimal places, which was considered sufficient to distinguish the relative differences among the investigated CSSs without implying excessive numerical precision.

The simplified net reaction describing the CO_2_-triggered protonation/bicarbonate-formation process is as follows:

(4) $$R_{3}N+{\mathrm{CO}}_{2}+H_{2}O\to [R_{3}\mathrm{NH}{]}^{+}+[{\mathrm{HCO}}_{3}{]}^{-}$$

The corresponding Gibbs free energy change is hereafter referred to as the Gibbs free energy of CO_2_-triggered protonation. The physical extraction process itself is not treated as a chemical reaction; rather, the calculated ΔG characterizes the chemical switching step that promotes the hydrophobic-to-hydrophilic conversion and subsequent phase partitioning of the solvent.

Gibbs free energy of the extraction reaction (∆G):

(5) $$∆G\;=\;G([R_{3}\mathrm{NH}{]}^{+}+\left[{HCO}_{3}{]}^{-}\right)-G(R_{3}N+{\mathrm{CO}}_{2}+H_{2}O)$$

Rate of change in extraction efficiency:

(6) $$\varphi \;=\;({∆G}_{0}-{∆G}_{1})/{∆G}_{0}$$

where ${∆G}_{0}$ represents the ΔG of the extraction reaction for the original CSSs (No. 1–15) and ${∆G}_{1}$ represents the ΔG of the corresponding modified molecules. Under constant temperature and pressure, ∆G < 0 indicates that the reaction is thermodynamically spontaneous, and ∆G > 0 indicates that the reaction is non-spontaneous.

When comparing a series of CSSs, a larger absolute value of ΔG (|ΔG|) corresponds to a greater thermodynamic driving force for the reaction. From an energetic perspective, this indicates a stronger response of CSSs to CO_2_ and a greater tendency to undergo polarity reversal [5].

#### 3.3.2. Evaluation of the Recovery Efficiency of CSSs

##### Establishment of Model Solvent Environments for Deprotonation and Recovery Analysis

In conventional SHS processes, the hydrophobic neutral amine is converted to a hydrophilic protonated form in the presence of CO_2_ and water, allowing transfer into an aqueous phase. Subsequent removal of CO_2_ by heating or gas stripping shifts the equilibrium toward the neutral amine, leading to deprotonation, phase separation, and solvent recovery [20,21]. Accordingly, water was selected in this study as the benchmark solvent environment for evaluating recovery-related thermodynamics.

Ethanol was introduced as an additional comparative polar protic environment rather than as a conventional industrial SHS regeneration solvent. The purpose of including ethanol was to evaluate the sensitivity of the calculated deprotonation thermodynamics to solvent polarity and solvation. Experimental studies have demonstrated that the dissociation constants and thermodynamic properties of protonated amines vary substantially between aqueous and alcohol-containing environments, including ethanol–water systems [78]. Moreover, CO_2_-responsive systems containing a tertiary-amine SHS, ethanol, and water have been experimentally demonstrated [21].

Therefore, the default solvation parameters for water and ethanol in Gaussian were employed as two comparative polar solvent models. The calculated ΔG values are interpreted as relative descriptors of the thermodynamic favorability of the deprotonation/regeneration process under different solvation conditions, rather than as direct predictions of experimentally measured solvent recovery efficiencies.

##### Evaluation of the Recovery Efficiency of CSSs—Deprotonation Process

The influence of molecular structure on the recovery behavior of 15 CSSs was preliminarily evaluated by comparing the ΔG associated with them and were further evaluated by calculating the ΔG associated with the deprotonation/regeneration processes in water and ethanol model solvent environments. The change in ΔG before and after structural modification was used to characterize the change rate in recovery efficiency, thereby quantitatively assessing the effects of different modification strategies on the recovery efficiency of CSSs.

The general equation describing the solvent recovery reaction is as follows:

(7) $${[R_{3}\mathrm{NH}{]}^{+}+[{\mathrm{HCO}}_{3}{]}^{-}\to R}_{3}N+{\mathrm{CO}}_{2}+H_{2}O$$

Recovery of Gibbs free energy ($∆G^{\prime }$):

(8) $$∆G^{\prime }=G\left(R_{3}N+{\mathrm{CO}}_{2}+H_{2}O\right)-G([R_{3}\mathrm{NH}{]}^{+}+\left[{HCO}_{3}{]}^{-}\right)$$

Recovery efficiency change rate:

(9) $$\varphi^{\prime }=({∆G}_{0}^{\prime }-{∆G}_{1}^{\prime })/{∆G}_{0}^{\prime }$$

where ${∆G}_{0}^{\prime }$ represents the Gibbs free energy of the recovery reaction for CSS molecules numbered 1–15 and ${∆G}_{1}^{\prime }$ represents the Gibbs free energy of the recovery reaction for modified molecules with corresponding codes.

### 3.4. Extraction Efficiency Analysis by a Linear Mixed Effects Model Approach

The linear mixed-effects model (LMM), enabling the joint evaluation of overall average effects and between-group heterogeneity [79,80], was used in this study to analyze the extraction efficiency of modified CSSs. Because multiple observations derived from the same parent molecule under different modification conditions are not fully independent, and because different parent molecules possess inherent structural differences, an LMM was constructed using the Statsmodels 0.14.0 package in Python 3.10. In this model, the extraction efficiency change rate of CSSs was specified as the response variable. Modification sites (i.e., C2, C3, and C4) and modification types (i.e., OH, phenyl, ester, and combined modification) were included as fixed effects, whereas the parent-molecule identifier was treated as a random effect to account for differences in the baseline response levels among parent molecules. The model formula is given as follows:

(10) $$Y_{ij}=\beta_{0}+\beta_{1}X_{1ij}+\beta_{2}X_{2ij}+u_{j}+\varepsilon_{ij}$$

where $Y_{ij}$ represents the extraction efficiency change rate of the (j)-th parent molecule under the (i)-th modification condition; $\beta_{0}$ denotes the average response level under the baseline condition; $X_{1ij}$ and $X_{2ij}$ represent the modification site (C2, C3, and C4) and modification type (OH, phenyl, ester, and combined modification), respectively; $u_{j}$ denotes the random intercept associated with the parent molecule; and $\varepsilon_{ij}$ represents the residual error term.

### 3.5. Thermodynamic Mechanism Analysis of Extraction Efficiency of Modified CSSs—A Support Vector Regression–Principal Component Analysis Approach

To elucidate the structure–activity relationship between the ΔG of CO_2_-switching reactions and the molecular structures of CSSs, the ΔG values obtained from Gaussian calculations were used as the target variable, while molecular descriptors were used as input features. A quantitative structure–activity relationship (QSAR) model was constructed to characterize the thermodynamic effects of CO_2_-switching reactions. Considering the high dimensionality of the original descriptor set, the potential for strong collinearity among variables, and the possible nonlinear relationship between ΔG and molecular structure, support vector regression (SVR) coupled with principal component analysis (PCA) was employed for model development [81].

First, 1444 molecular descriptors were calculated for the CSSs using PaDEL-Descriptor. The original dataset was then rigorously preprocessed by removing all-zero features, samples containing missing values, low-variance features with a non-zero proportion below 5%, and highly collinear features with correlation coefficients greater than 0.95. Subsequently, outliers were identified and removed based on the three-standard-deviation criterion for ΔG and Huber regression analysis. The retained features were standardized using Z-score normalization, and a Box–Cox transformation was applied to the positive ΔG values to satisfy model assumptions. Next, the mutual information method was used to select the 15 most informative features, and second-order interaction terms were generated from these variables. PCA was then performed on the standardized feature set, retaining principal components that preserved 99% of the total information. Finally, an SVR model was constructed using the retained principal components as input variables and the transformed ΔG values as the output variable. The radial basis function (RBF) was selected as the kernel function, and the optimal combination of the penalty parameter (C), kernel parameter (γ), and error tolerance parameter (ε) was determined through grid-search optimization. Model efficiency was comprehensively evaluated using the coefficient of determination (R2), root mean square error (RMSE), and mean absolute percentage error (MAPE) [33]. In addition, residual analysis was performed to assess model stability, and permutation importance analysis was employed to quantify the contributions of individual principal components. These analyses further facilitated the interpretation of the key molecular structural factors governing the thermodynamic effects of CO_2_-switching reactions.

### 3.6. Assessment of Environmental and Human Health Toxicity of Novel CSSs Using ADMETlab 3.0

Toxicity prediction and evaluation of the effective novel CSSs designed in this study were performed using the ADMETlab 3.0 (https://admetlab3.scbdd.com/) online platform [82]. The evaluation procedure consisted of two steps. First, the molecular structures of all target compounds were converted into Simplified Molecular Input Line Entry System (SMILES) format. Subsequently, the SMILES strings were either submitted individually to the ADMET Evaluation module for single-compound analysis or uploaded as a comma-separated values (CSV) file to the ADMET Screening module for batch prediction.

To assess the potential impacts of the effective novel CSSs on human health and the environment, 24 toxicity-related endpoints were selected from the prediction results for further analysis. These endpoints included, but were not limited to, Skin Sensitization, Eye Corrosion/Irritation, Respiratory Toxicity, IGC50, LC50FM, and LC50DM. Toxicity predictions were reported as either classification probabilities (ranging from 0 to 1) or regression-based estimates (e.g., LD50, mg kg^−1^), together with uncertainty scores that were used to evaluate the reliability of the predictions. Based on these results, the toxicity risk levels of the effective novel CSSs were classified and compared.

## 4. Conclusions

This study established an integrated computational framework combining DFT-based thermodynamic analysis, systematic molecular modification, linear mixed-effects modeling, machine learning, recovery assessment, and toxicity prediction for the rational design of CO2-switchable solvents. The results demonstrate that hydrophobic structure, modification position, and functional-group type jointly govern the thermodynamic and extraction responses of CSSs, highlighting the importance of considering their interactions rather than optimizing individual structural factors independently. The framework enabled the identification of promising CSS candidates with enhanced extraction performance while maintaining favorable recovery characteristics and predicted safety profiles. Beyond the specific CSSs investigated here, this structure-guided strategy provides a transferable computational approach for elucidating structure–performance relationships and prioritizing candidate switchable solvents before experimental evaluation. The identified candidates therefore provide a theoretical basis for further experimental validation and may support the development of more efficient and potentially sustainable solvent systems for oil-based drilling-cuttings treatment and resource recovery.

## Figures and Tables

**Figure 1:**
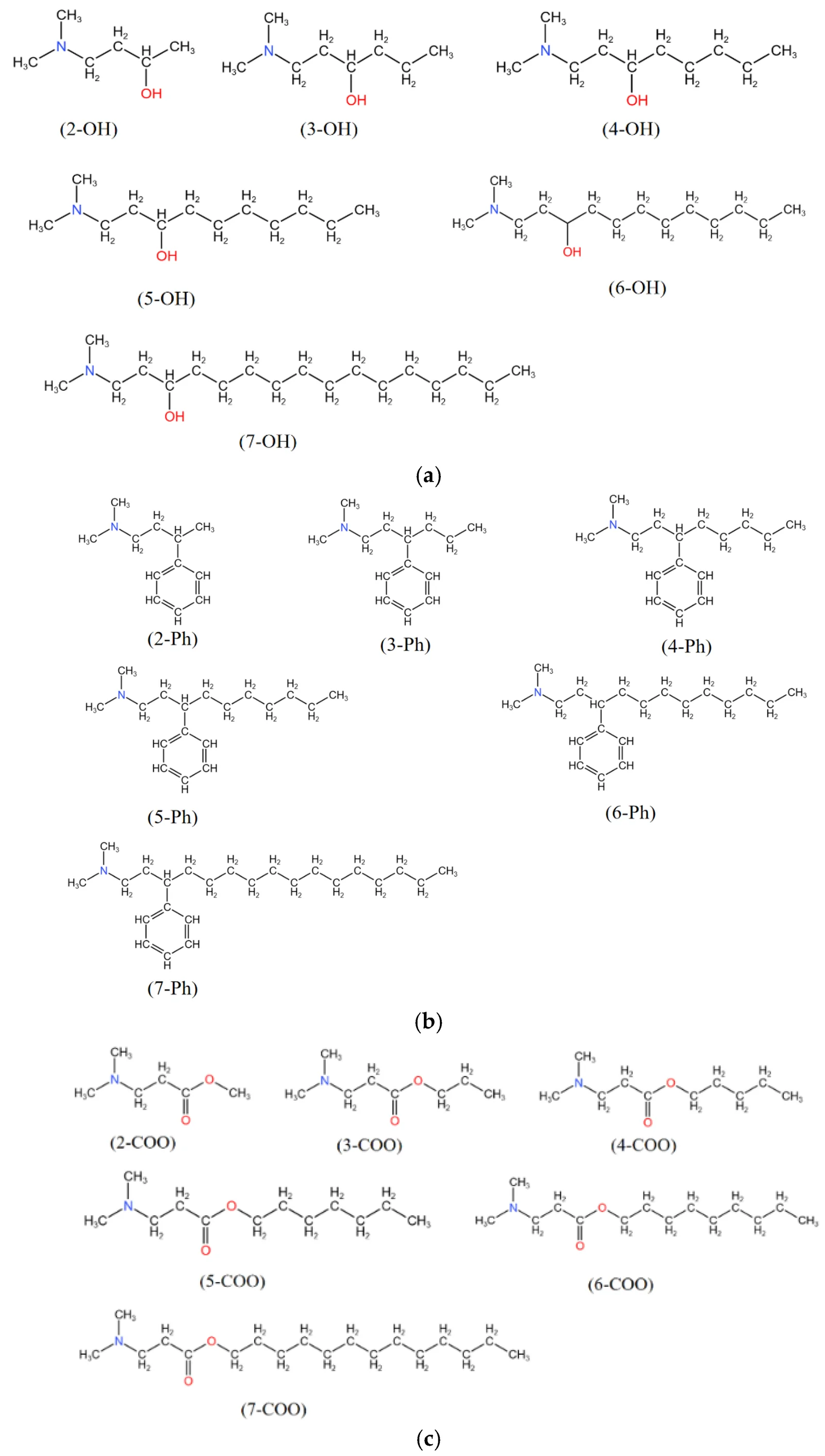

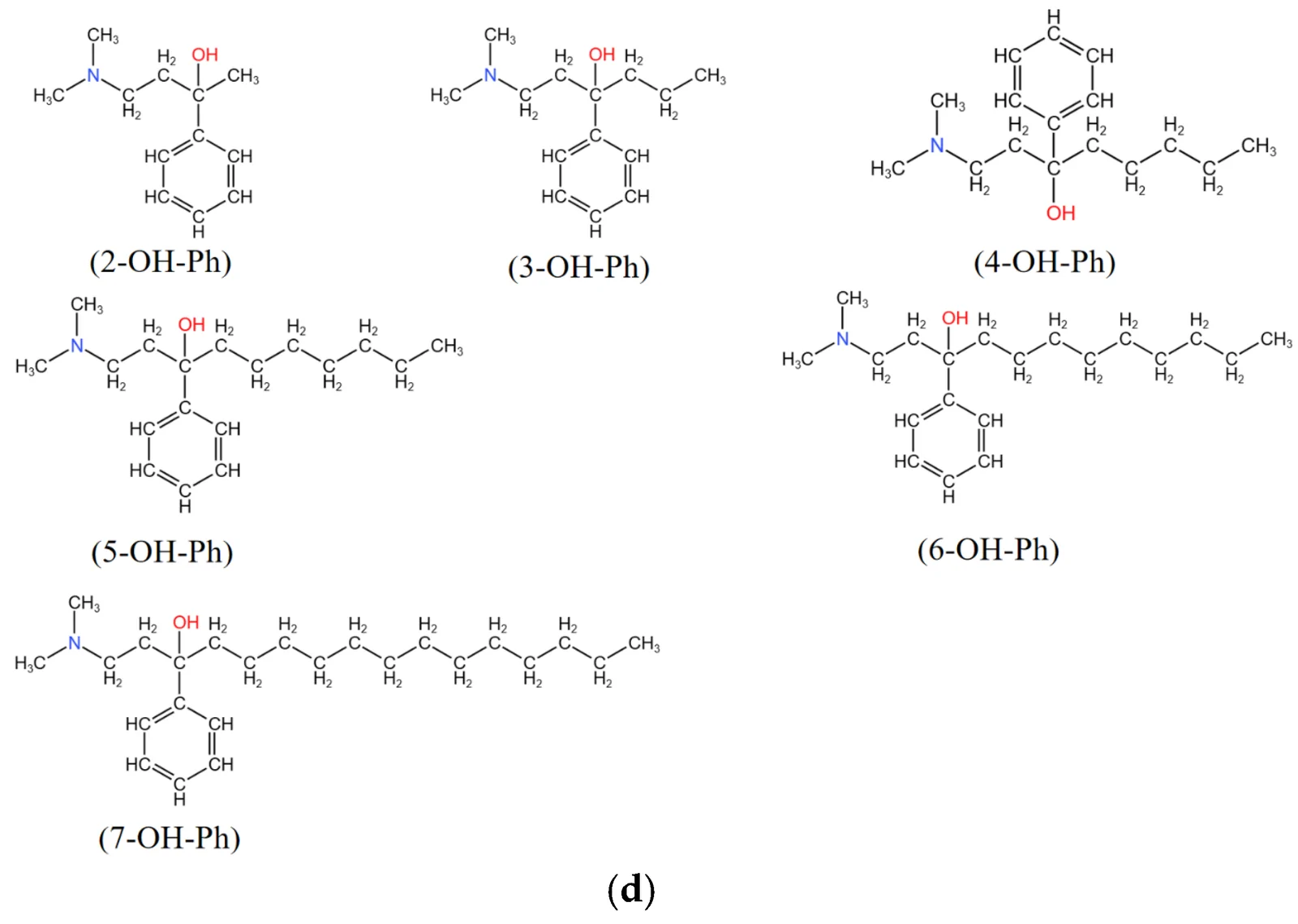
Structures of novel CO_2_-switchable solvents derived from No. 2–7 at the C3 position. (Note: Molecular labels are coded as modification site–parent molecule number–modification strategy. The C8-equivalent hydrophobic tail, hydroxyl-group, phenyl-group, and ester-group modifications are abbreviated as –C, -OH, -Ph, and -COO, respectively.) (**a**) Molecular structures of No. 2–7 CSSs modified by modified with hydroxyl groups. (**b**) Molecular structures of No. 2–7 CSSs modified by phenyl groups. (**c**) Molecular structures of No. 2–7 CSSs modified by ester groups. (**d**) Molecular structures of No. 2–7 CSSs modified by both hydroxyl and phenyl groups.

**Figure 2:**
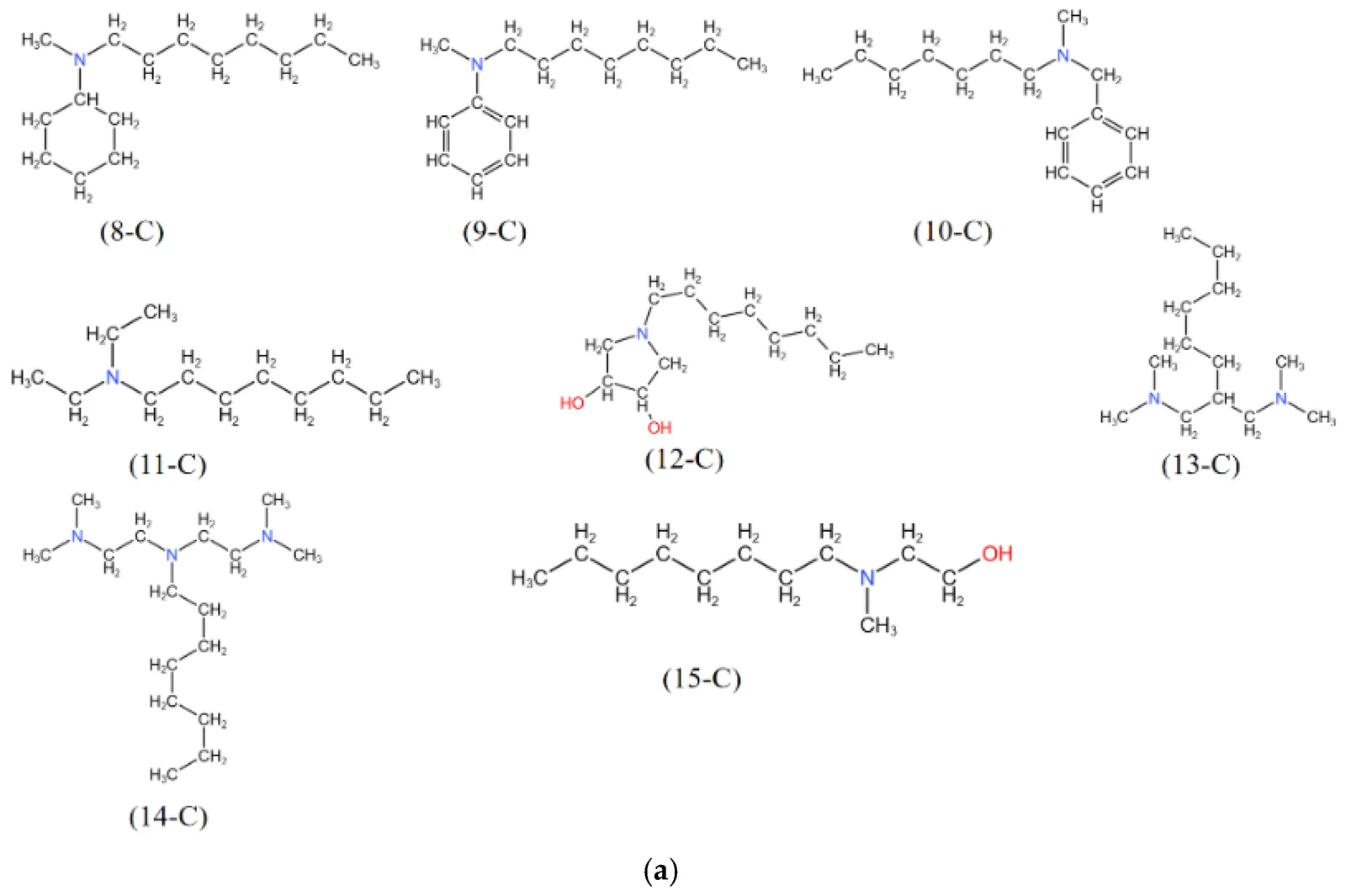

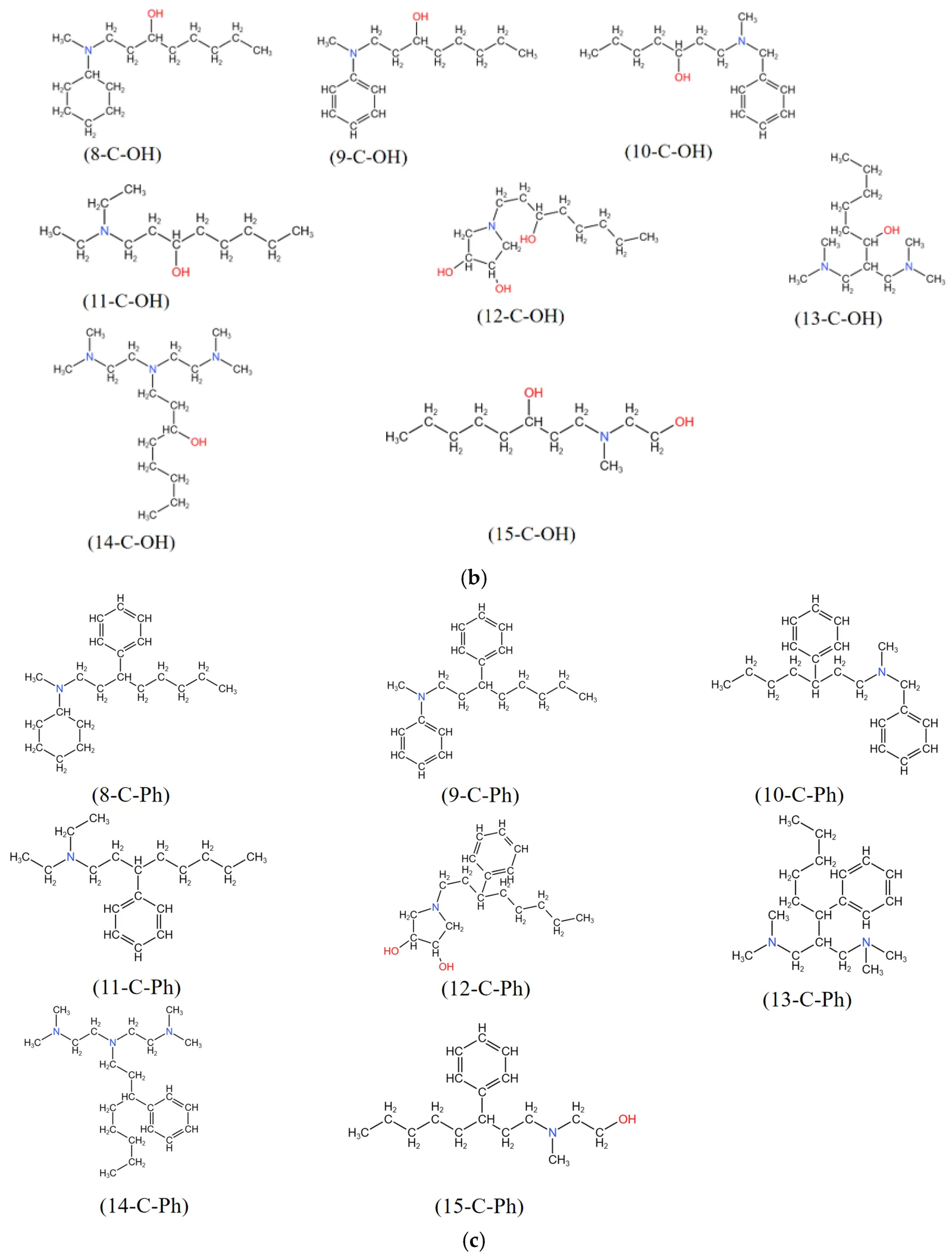

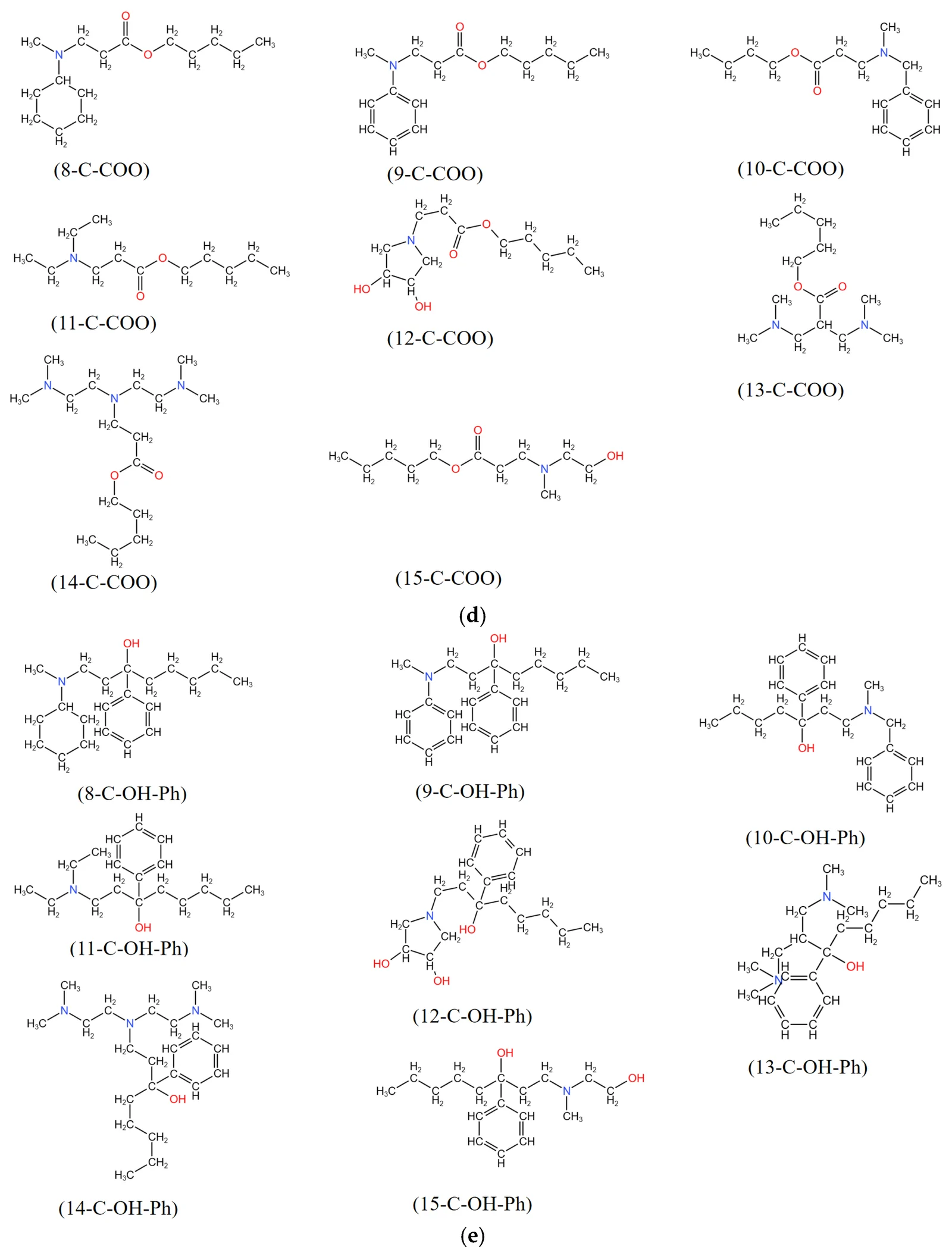
Structures of novel CSSs derived from No. 8–15 with functional-group modifications at the C3 position. (**a**) Molecular structures of No. 8–15 CSSs modified by C_8_-equivalent hydrophobic tails. (**b**) Molecular structures of No. 8–15 CSSs modified by C8-equivalent hydrophobic tails and hydroxyl group. (**c**) Molecular structures of No. 8–15 CSSs modified by C_8_-equivalent hydrophobic tails and phenyl groups. (**d**) Molecular structures of No. 8–15 CSSs modified by C_8_-equivalent hydrophobic tails and ester groups. (**e**) Molecular structures of No. 8–15 CSSs modified by C_8_-equivalent hydrophobic tails and both hydroxyl and phenyl groups.

**Figure 3:**
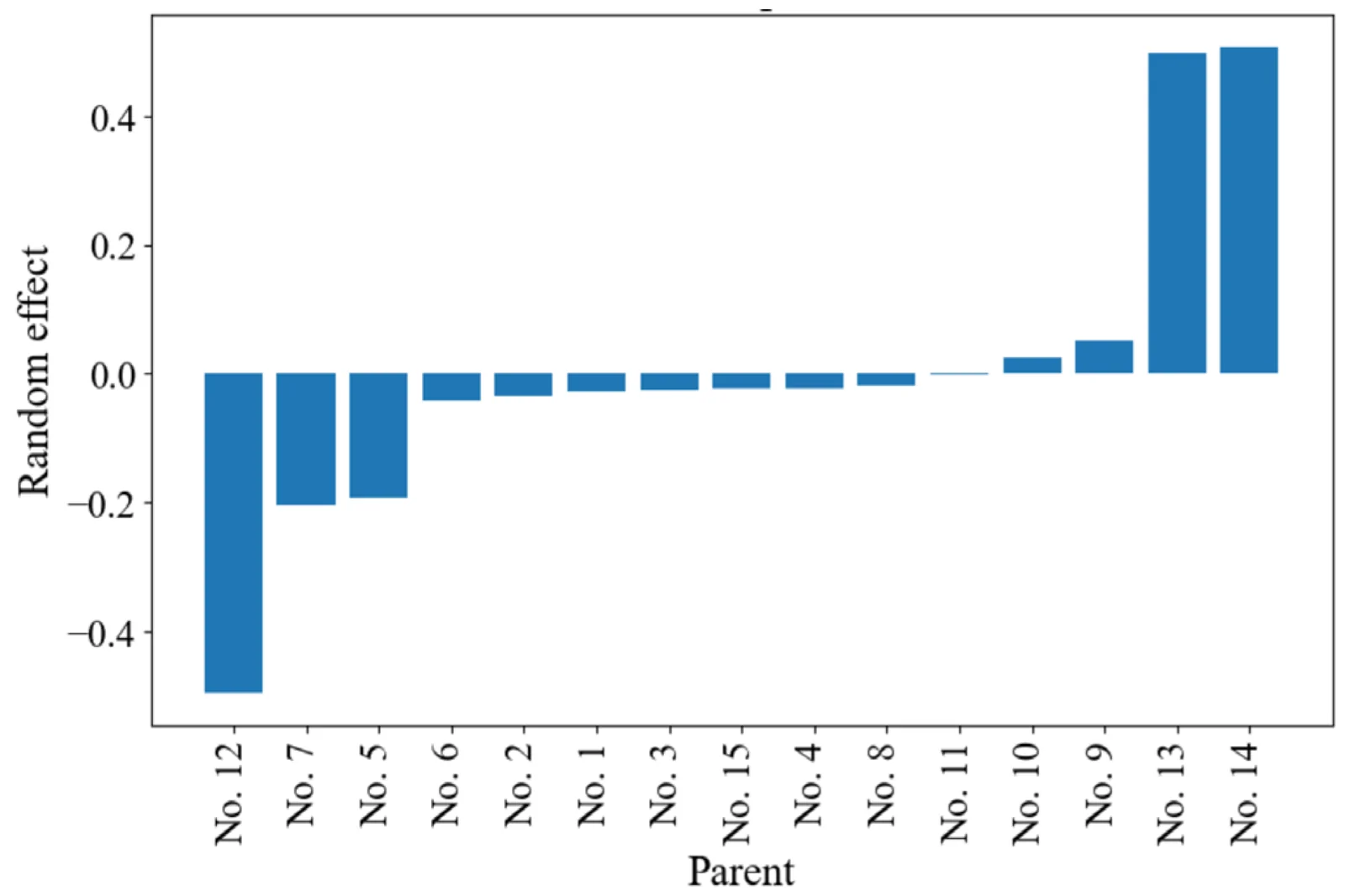
Random effects of 15 CSSs.

**Figure 4:**
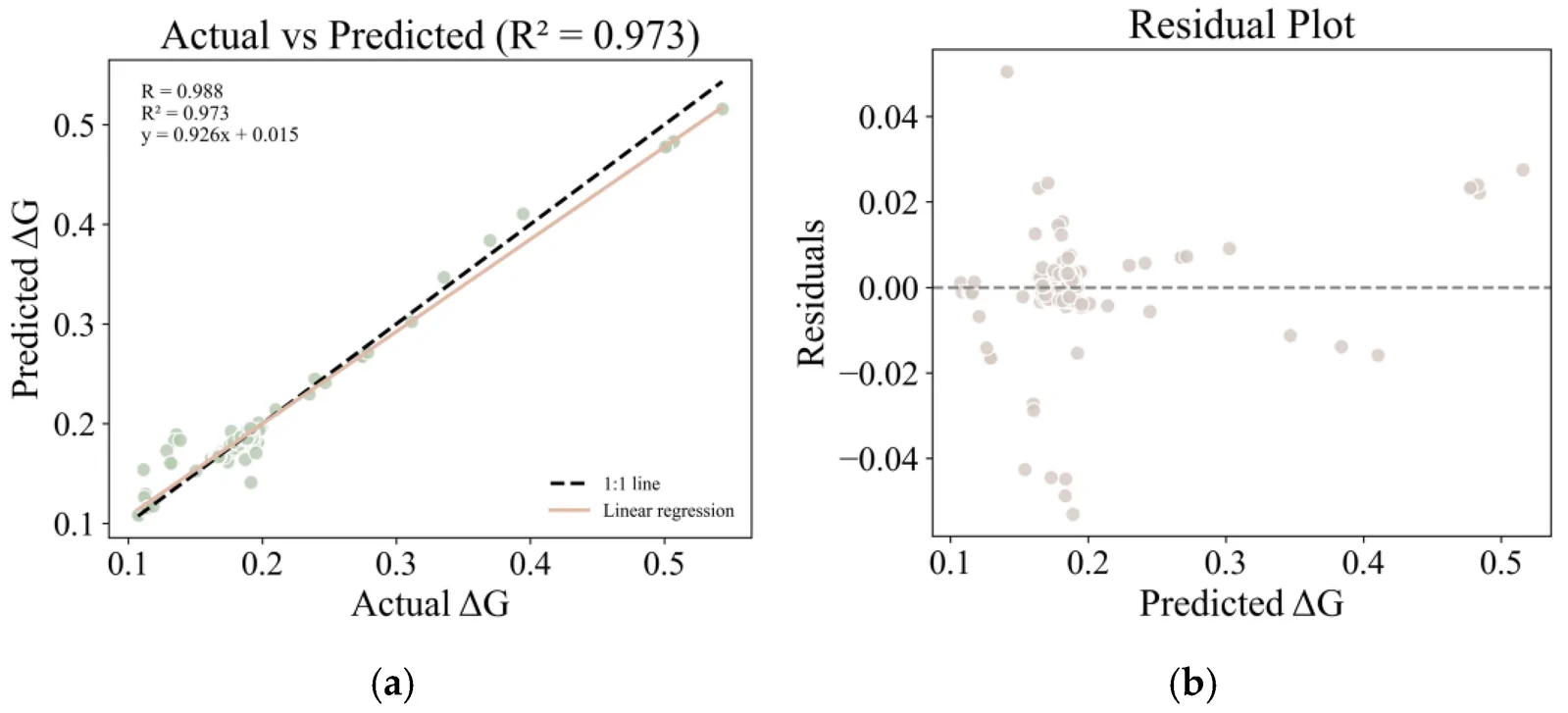
Scatter plot of the predicted versus actual ΔG values for the SVR-PCA model (**a**) and the corresponding residual plot (**b**).

**Figure 5:**
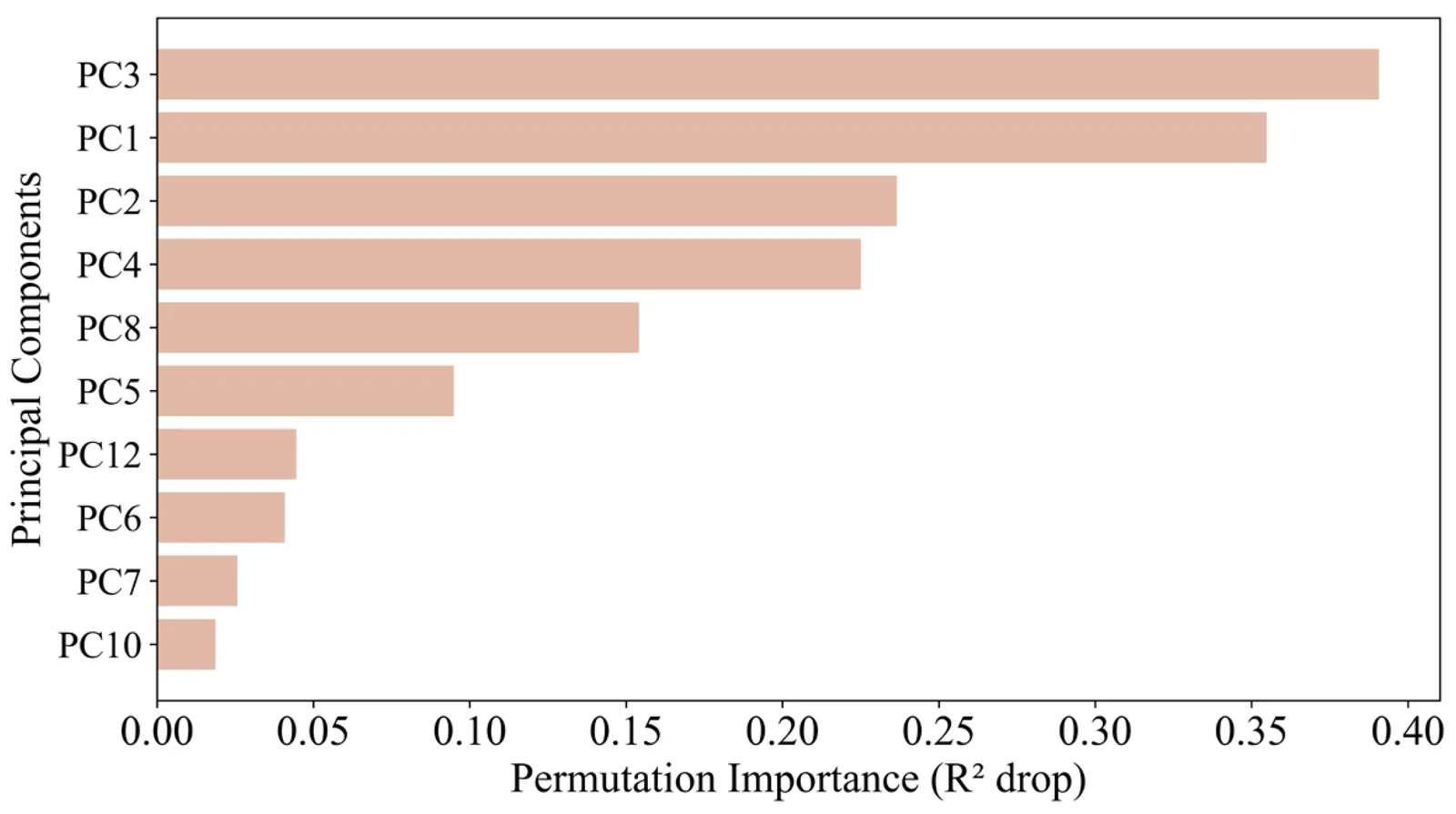
Permutation importance of the principal components in the PCA-SVR model.

**Figure 6:**
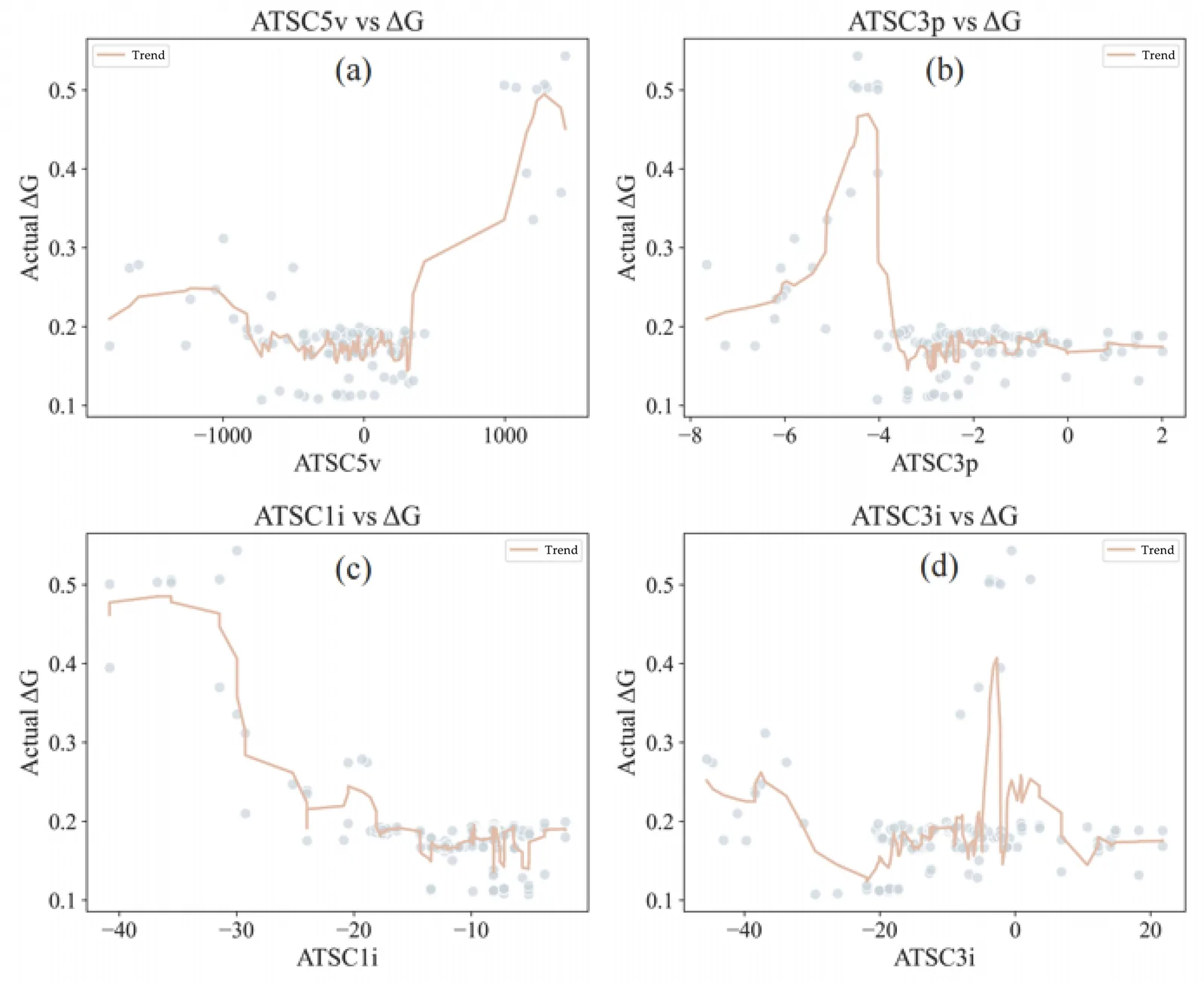
Relationships between ΔG and the four most influential molecular descriptors, ATSC5v (**a**), ATSC3p (**b**), ATSC1i (**c**) and ATSC3i (**d**).

**Figure 7:**
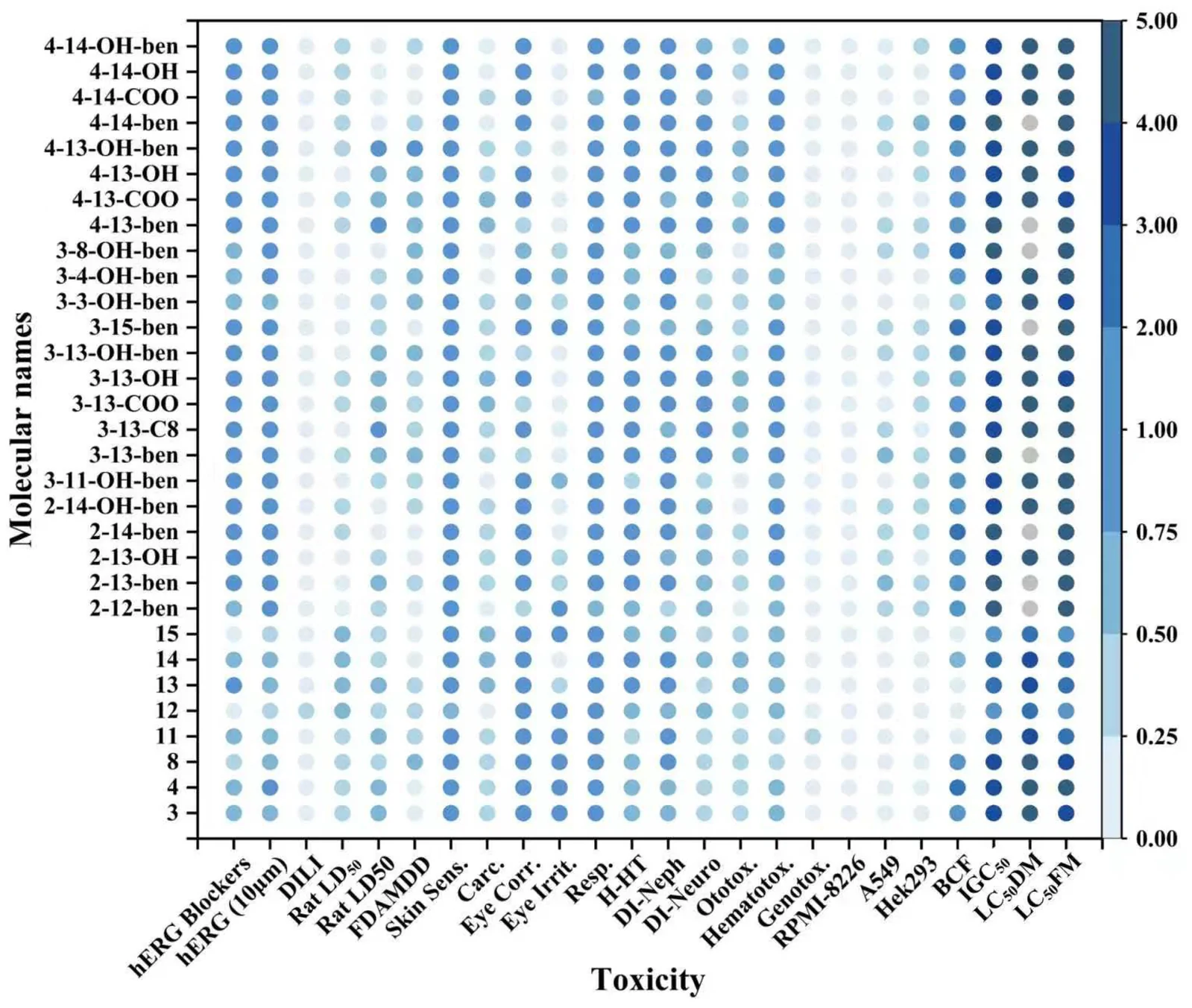
Heatmap of predicted toxicity profiles of the novel CSSs. (The toxicity score ranges from 0 to 5, with lower scores indicating lower predicted toxicity and higher scores indicating higher predicted toxicity).

**Figure 8:**
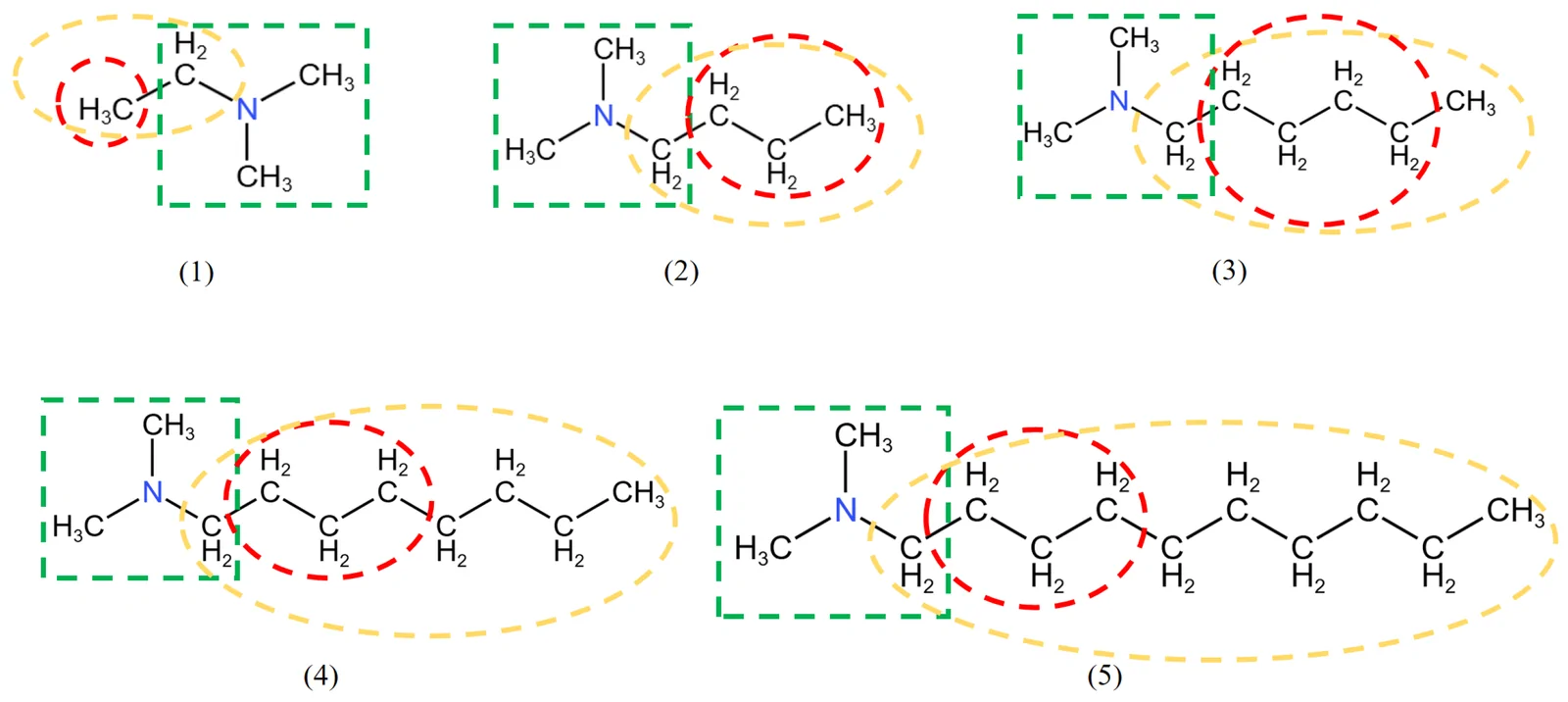

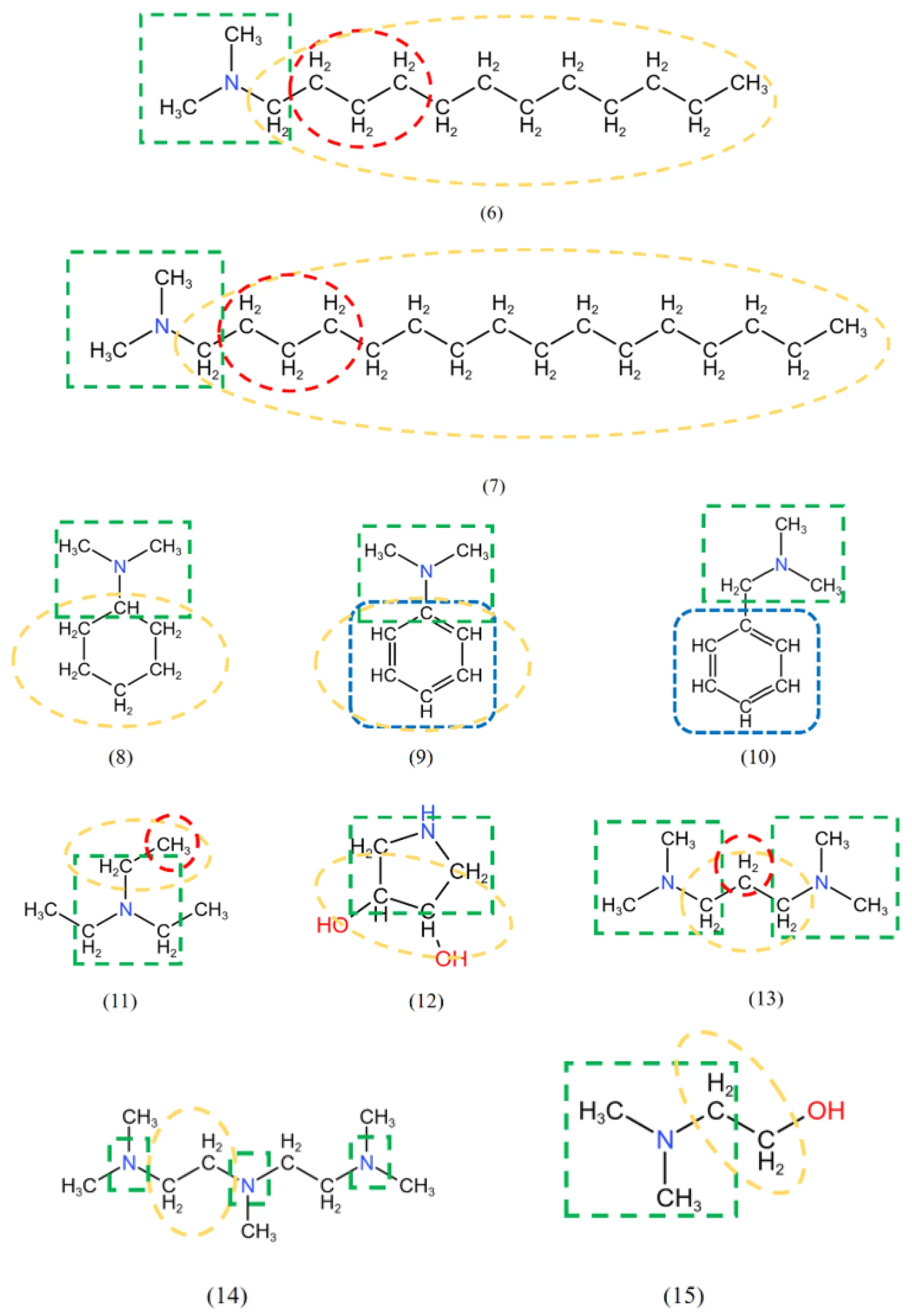
Molecular structure of 15 CO_2_ switchable solvents. (Note: Yellow represents the hydrophobic tail region, green represents the central linker region, red represents the hydrophobic linker region, and blue represents the heteroaromatic region).

**Figure 9:**
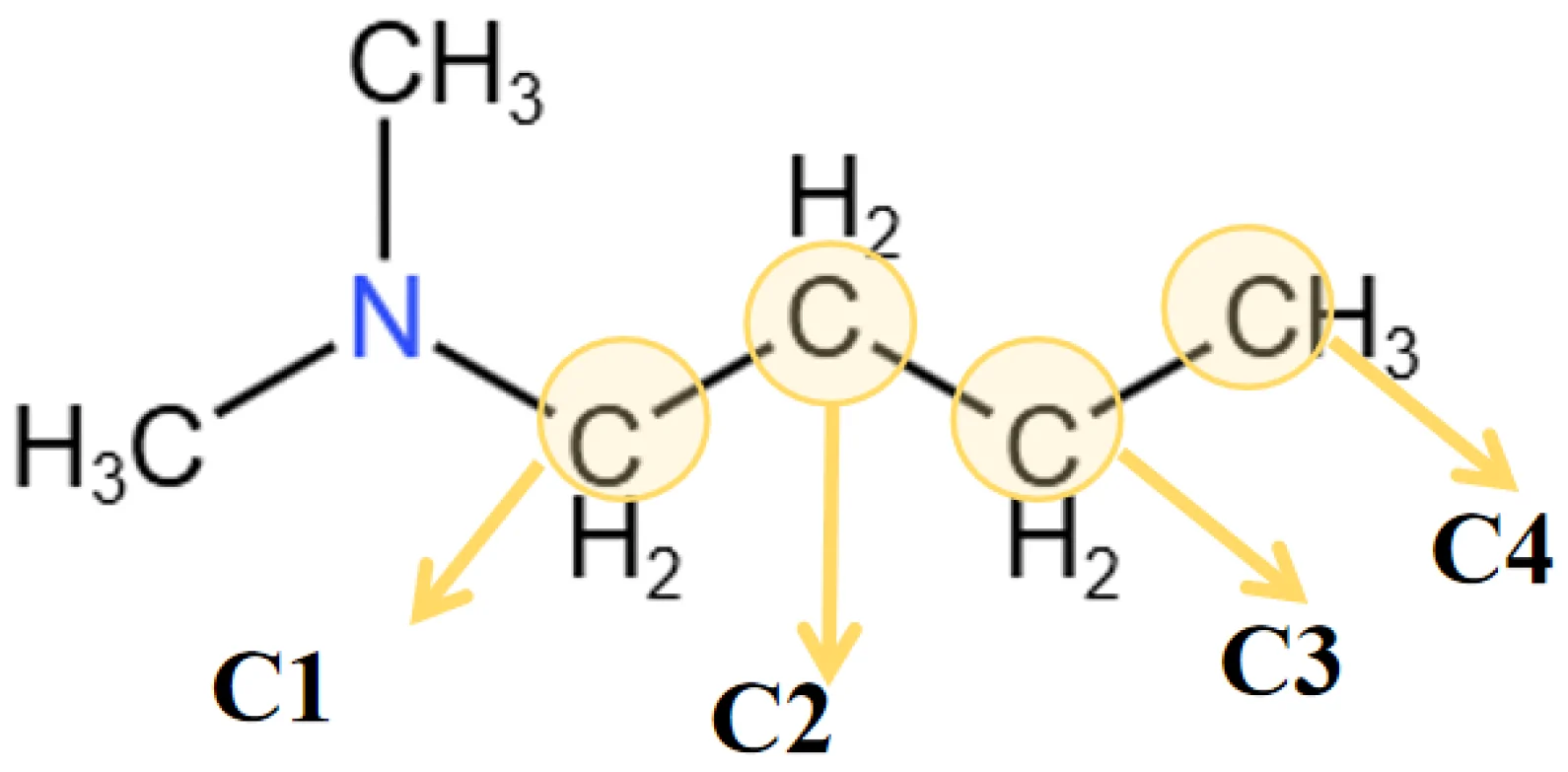
The modified sites of CSS N,N-dimethylbutylamine (No. 2).

**Table 1:** Standard Gibbs free energy changes (ΔG°) of CO_2_-switching reactions for the 15 CSSs in a petroleum-like solvent environment.

No.	Structure Chart	Reactant	Product	ΔG_0_	ΔG° (kcal/mol)
1	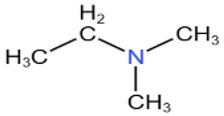	−213.672652	−214.176368	0.166450	104.50
2	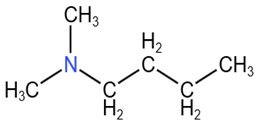	−292.248844	−292.752249	0.166761	104.70
3	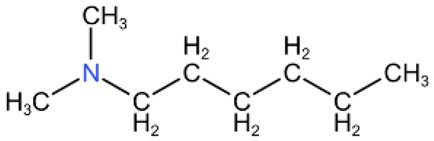	−370.824495	−371.328288	0.166373	104.42
4	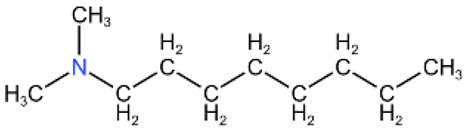	−449.400448	−449.904224	0.166390	104.43
5	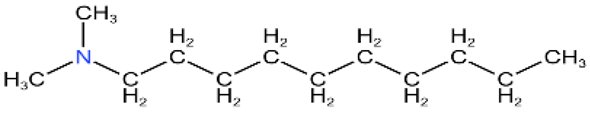	−527.977781	−528.480767	0.167180	104.93
6	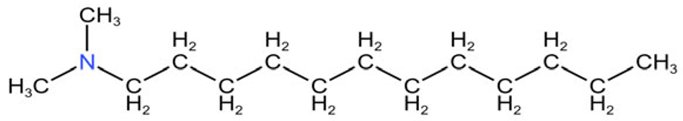	−606.552648	−607.057372	0.165442	103.81
7	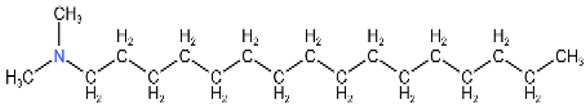	−763.705163	−764.210624	0.164705	103.35
8	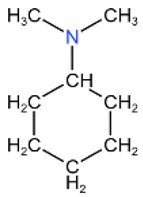	−369.633501	−370.137794	0.165873	104.09
9	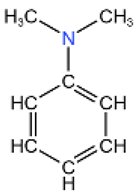	−366.083664	−366.559763	0.194067	121.82
10	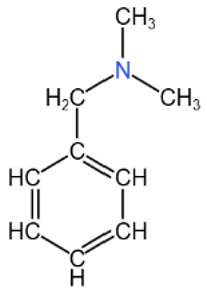	−405.36644	−405.922488	0.114118	71.60
11	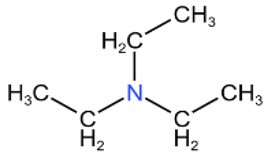	−292.248864	−292.748869	0.170161	106.81
12	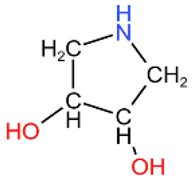	−362.898803	−363.404974	0.163995	102.93
13	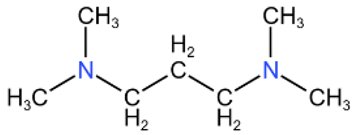	−386.851596	−386.851596	1.340332	841.31
14	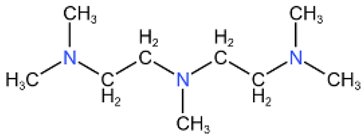	−520.743749	−522.250850	0.503397	315.99
15	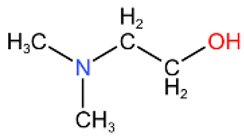	−288.873992	−289.378178	0.165980	104.12

Note: ΔG° represents the standard Gibbs free energy change in the CO_2_-switching reaction. The values in kcal/mol were converted from Hartree using 1 Hartree = 627.5095 kcal/mol.

**Table 2:** Extraction efficiency change rates of novel CSSs.

	Extraction Efficiency Change Rate (*φ*)											
		C2 Position			C3 Position				C4 Position			
No.	C_8_	Hydroxyl-Group Modification	Phenyl-Group Modification	Ester-Group Modification	Hydroxyl-Group Modification	Phenyl-Group Modification	Ester-Group Modification	Combined Hydroxyl- and Phenyl-Group Modification	Hydroxyl-Group Modification	Phenyl-Group Modification	Ester-Group Modification	Combined Hydroxyl- and Phenyl-Group Modification
1	—	−0.36%	−14.82%	−4.59%	—	—	—	—	—	—	—	—
2	—	0.88%	−7.38%	−13.14%	−0.24%	−14.17%	−12.36%	−13.88%	−0.38%	−17.04%	—	−14.84%
3	—	−2.05%	−7.36%	−18.16%	−0.38%	−14.52%	−11.55%	19.19%	0.25%	−15.24%	−15.05%	−14.04%
4	—	1.37%	−6.87%	−8.34%	−0.87%	−13.48%	−12.96%	16.66%	−0.20%	−15.97%	−15.79%	−15.47%
5	—	−1.16%	−6.54%	−6.37%	−0.24%	−300.86%	−11.84%	−12.88%	0.63%	−14.38%	−10.30%	−13.34%
6	—	−0.53%	−8.38%	−8.73%	−0.39%	−14.05%	−12.48%	−12.52%	−3.51%	−15.45%	−14.34%	−14.46%
7	—	0.57%	−5.73%	−8.14%	−1.55%	−306.89%	−11.75%	−15.24%	−0.45%	−17.21%	−15.49%	−15.34%
8	−1.10%	−1.78%	−6.13%	−9.09%	−4.74%	−13.85%	−10.99%	22.56%	−0.69%	−13.37%	−12.98%	−13.51%
9	7.58%	8.28%	3.58%	3.53%	0.65%	5.69%	7.50%	6.15%	7.66%	5.99%	7.96%	5.18%
10	3.59%	2.38%	5.04%	5.96%	1.09%	0.11%	1.74%	0.78%	1.92%	−0.50%	−0.13%	−3.78%
11	1.35%	4.84%	−4.10%	−13.20%	−3.02%	1.00%	−8.76%	22.68%	1.16%	−10.54%	−11.05%	−10.76%
12	−1.19%	−2.30%	19.07%	−21.50%	−0.73%	−16.41%	−14.63%	−9.57%	−1.39%	−20.47%	8.41%	−861.10%
13	81.57%	86.91%	79.53%	—	82.16%	85.29%	84.35%	79.50%	82.48%	86.86%	76.75%	79.21%
14	0.05%	−0.55%	26.51%	33.36%	0.18%	−0.69%	0.51%	−7.92%	262.83%	278.43%	21.61%	267.86%
15	−0.78%	−0.17%	−7.68%	−12.67%	−1.13%	18.10%	−13.38%	−17.09%	−0.76%	−6.51%	−16.01%	−15.18%

**Table 3:** Mean effect matrix for modification site–functional group interactions.

Mod-Type Site	Phenyl	Combination	Ester	OH
2-C	0.039	−0.058	NaN	0.064
3-C	−0.418	0.056	−0.190	0.051
4-C	0.160	−0.457	0.003	0.250

**Table 4:** Recovery-related thermodynamic change rates of the selected CSS candidates in water and ethanol model solvent environments.

Modifying Molecules	Logkow	Recovery Efficiency Change Rates	
		Ethanol	Water
3-3-OH-Ph	2.63	−3.29%	2.91%
3-4-OH-Ph	3.61	−2.83%	−2.80%
3-8-C-OH-Ph	5.88	−3.74%	−3.73%
3-11-C-OH-Ph	4.59	−4.04%	−8.85%
2-12-C-Ph	3.09	−3.09%	−3.32%
2-13-C-OH	1.6	−14.08%	−14.50%
2-13-C-Ph	4.77	−0.07%	−0.31%
3-13-C-Ph	4.73	−8.51%	−8.85%
3-13-C-COO	1.66	−5.31%	−7.47%
3-13-C-OH-Ph	3.23	−0.23%	−0.53%
3-13-C-OH	1.56	−4.24%	−4.18%
13-C	3.1	−0.15%	−1.00%
4-13-C-OH	1.56	−5.64%	−3.12%
4-13-C-Ph	4.73	−11.62%	−11.63%
4-13-C-COO	1.66	2.11%	3.23%
4-13-C-OH-Ph	3.23	−1.42%	−1.43%
2-14-C-Ph	4.28	−2.99%	−4.40%
2-14-C-OH-Ph	2.78	−19.24%	−20.32%
4-14-C-OH	1.33	0.04%	0.04%
4-14-C-Ph	4.51	−0.04%	−0.04%
4-14-C-COO	1.43	2.20%	2.28%
4-14-C-OH-Ph	3	−3.09%	−2.98%
3-15-C-Ph	4.14	−3.03%	3.46%

**Table 5:** Molecular information and classification of 15 amine-based parent compounds used for CO_2_-switchable solvent design.

Number	Names	Molecular Formula	CAS Numbers	Category
1	N,N-dimethylethylamine	C_4_H_11_N	598-56-1	CO_2_-responsive reference amine [54]
2	N,N-dimethylbutylamine	C_6_H_15_N	927-62-8	Reported SHS [53]
3	N,N-dimethylhexylamine	C_8_H_19_N	4385-04-0	Reported SHS [15]
4	N,N-dimethyloctylamine	C_10_H_23_N	7378-99-6	Reported SHS [55]
5	N,N-dimethyldecylamine	C_12_H_27_N	1120-24-7	CO_2_-responsive reference amine [56]
6	N,N-dimethyldodecylamine	C_14_H_31_N	112-18-5	CO_2_-responsive reference amine [57]
7	N,N-dimethylhexadecylamine	C_18_H_39_N	112-69-6	Reported SHS [58]
8	N,N-dimethylcyclohexylamine	C_8_H_17_N	98-94-2	Reported SHS [51]
9	N,N-dimethylaniline	C_8_H_11_N	121-69-7	Reported SHS [59]
10	N,N-dimethylbenzylamine	C_9_H_13_N	103-83-3	Reported SHS [15]
11	Triethylamine	C_6_H_15_N	121-44-8	Reported SHS [15]
12	Polyetheramine D 230	C_4_H_9_NO_2_	Nan	CO_2_-responsive reference amine [60]
13	Tetramethylpropylenediamine	C_7_H_18_N_2_	110-95-2	CO_2_-responsive reference amine [61]
14	Pentamethyldiethylenetriamine	C_9_H_23_N_3_	3030-47-5	CO_2_-responsive reference amine [62]
15	N,N-dimethylethanolamine	C_4_H_11_NO	108-01-0	CO_2_-responsive reference amine [63]

**Table 6:** Solvent environment parameters for equivalent crude oil (Arab Light crude).

SARAs Fractions	Proportion (wt%)	Relative Permittivity (ε)	High-Freq. Dielectric Constant ε∞ (=n225)	H-Bond Acceptor Basicity (β)	Surface Tension (γ) (mN·m^−1^)	Aromatic Carbon Fraction (fA)
Saturates	59.9	2.0060	1.4151	0.00	35.85	0.000
Aromatics	35.8	2.3741	1.4936	0.14	40.2	0.857
Resins	1.54	12.978	1.5073	0.52	52.62	0.833
Asphaltenes	2.77	2.3741	1.4936	0.14	40.2	0.857

## Data Availability

The data presented in this study are available on request from the corresponding author. The data are not publicly available due to privacy.

## Supplementary Materials

The following supporting information can be downloaded at: https://www.mdpi.com/article/10.3390/molecules31193444/s1, Table S1: Gibbs free energies of CO_2_, H_2_O, and HCO_3_^−^ in the petroleum-equivalent solvent environment; Table S2: Changes in extraction reaction efficiency following C8-equivalent hydrophobic tail modification of CO_2_-switchable solvent molecules No. 8–15; Table S3: Changes in extraction reaction efficiency following hydroxyl group introduction at the C2 position of the hydrophobic linker region; Table S4: Changes in extraction reaction efficiency following phenyl group introduction at the C2 position of the hydrophobic linker region; Table S5: Changes in extraction reaction efficiency following combined hydroxyl/phenyl modification at the C2 position of the hydrophobic linker region; Table S6: Changes in extraction reaction efficiency following hydroxyl group introduction at the C3 position of the hydrophobic linker region; Table S7: Changes in extraction reaction efficiency following phenyl group introduction at the C3 position of the hydrophobic linker region; Table S8: Changes in extraction reaction efficiency following ester group introduction at the C3 position of the hydrophobic linker region; Table S9: Changes in extraction reaction efficiency following combined hydroxyl/phenyl modification at the C3 position of the hydrophobic linker region; Table S10: Changes in extraction reaction efficiency following hydroxyl group introduction at the C4 position of the hydrophobic linker region; Table S11: Changes in extraction reaction efficiency following phenyl group introduction at the C4 position of the hydrophobic linker region; Table S12: Changes in extraction reaction efficiency following ester group introduction at the C4 position of the hydrophobic linker region; Table S13: Changes in extraction reaction efficiency following combined hydroxyl/phenyl modification at the C4 position of the hydrophobic linker region; Table S14: Gibbs free energies of CO_2_, H_2_O, and HCO_3_^−^ in the solvent recovery environments; Table S15: Gibbs free energies of the recovery reactions of CO_2_-switchable solvent molecules in the solvent recovery environments; Table S16: Predicted values for 24 toxicity endpoints of CO_2_-switchable solvent molecules; Table S17: Structure guide and nomenclature of the selected modified CSS candidates.
