# Supplementary material for: Design of Highly Efficient CO2-Switchable Solvents Based on Extraction and Recovery Performance
Source: Molecules. 2026 Sep 27;31(19):3444. doi: 10.3390/molecules31193444 (PMC13666905; doi:10.3390/molecules31193444)
Supplement: Supplementary file 1 [file molecules-31-03444-s001.zip › molecules-4571468-supplementary.pdf]

Supplementary Materials for

Design of Highly Efficient CO_2-Switchable Solvents Based on Extraction and Recovery Performance

Wen Ren [1 2,†], Luanxiao Wei [3,†], Yisu Fang [3,†], Xingchun Li [2], Yu Li [3 *,] Chunmao Chen [1]

1 State Key Laboratory of Petroleum Pollution Control, China University of Petroleum-Beijing, Beijing 102200, China; renwen@cnpc.com.cn; c.chen@cup.edu.cn

2 State Key Laboratory of Petroleum Pollution Control, CNPC Research Institute of Safety and Environment Technology, Beijing 102206, China; renwen@cnpc.com.cn; li-xingchun@cnpc.com.cn

3 College of Environmental Science and Engineering, North China Electric Power University, Beijing, 102206, China; wlxiao2024@163.com; 120242232021@ncepu.edu.cn; 50201549@ncepu.edu.cn

* *Correspondence: 50201549@ncepu.edu.cn*

† These authors have contributed equally to the study and they receive equal credit.

This SM file contains: 13 Pages; 17 Tables.

Table S1. Gibbs free energies of CO_2, H_2O, and HCO_3^- in the petroleum-equivalent solvent environment

Table S2. Changes in extraction reaction efficiency following C_8-equivalent hydrophobic tail modification of CO_2-switchable solvent molecules No. 8-15

Table S3. Changes in extraction reaction efficiency following hydroxyl group introduction at the C2 position of the hydrophobic linker region

Table S4. Changes in extraction reaction efficiency following phenyl group introduction at the C2 position of the hydrophobic linker region

Table S5. Changes in extraction reaction efficiency following combined hydroxyl/phenyl modification at the C2 position of the hydrophobic linker region

Table S6. Changes in extraction reaction efficiency following hydroxyl group introduction at the C3 position of the hydrophobic linker region

Table S7. Changes in extraction reaction efficiency following phenyl group introduction at the C3 position of the hydrophobic linker region

Table S8. Changes in extraction reaction efficiency following ester group introduction at the C3 position of the hydrophobic linker region

Table S9. Changes in extraction reaction efficiency following combined hydroxyl/phenyl modification at the C3 position of the hydrophobic linker region

Table S10. Changes in extraction reaction efficiency following hydroxyl group introduction at the C4 position of the hydrophobic linker region

Table S11. Changes in extraction reaction efficiency following phenyl group introduction at the C4 position of the hydrophobic linker region

Table S12. Changes in extraction reaction efficiency following ester group introduction at the C4 position of the hydrophobic linker region

Table S13. Changes in extraction reaction efficiency following combined hydroxyl/phenyl modification at the C4 position of the hydrophobic linker region

Table S14. Gibbs free energies of CO_2, H_2O, and HCO_3^- in the solvent recovery environments

Table S15. Gibbs free energies of the recovery reactions of CO_2-switchable solvent molecules in the solvent recovery environments

Table S16. Predicted values for 24 toxicity endpoints of CO_2-switchable solvent molecules

Table S17. Structure guide and nomenclature of the selected modified CSS candidates.

Table S1. Gibbs free energies of CO_2, H_2O, and HCO_3^- in the petroleum-equivalent solvent environment

Material	CO_2(Hartree)	H_2O(Hartree)	HCO_3(Hartree)
Standard gibbs free energy	-188.586447	-76.414244	-264.330525

Table S2. Changes in extraction reaction efficiency following C_8-equivalent hydrophobic tail modification of CO_2-switchable solvent molecules No. 8-15

No.	Reactants	Products	ΔG_1	ΔG_0-ΔG_1	Effect change rate (φ)
8	-644.645065	-645.147539	0.167692	-0.001819	-1.10%
9	-641.098668	-641.589486	0.179348	0.014719	7.58%
10	-641.092737	-641.651663	0.111240	0.004137	3.59%
11	-527.971761	-528.474056	0.167871	0.002290	1.35%
12	-677.204763	-677.708987	0.165942	-0.001947	-1.19%
13	-622.570910	-623.664262	0.246980	1.093352	81.57%
14	-795.750094	-797.257451	0.503141	0.000256	0.05%
15	-563.892202	-564.395096	0.167272	-0.001292	-0.78%

Table S3. Changes in extraction reaction efficiency following hydroxyl group introduction at the C2 position of the hydrophobic linker region

No.	Reactants	Products	ΔG_1	ΔG_0-ΔG_1	Effect change rate (φ)
1	-288.876942	-289.380053	0.167055	-0.000605	-0.36%
2	-367.459117	-367.963984	0.165299	0.001462	0.88%
3	-446.036903	-446.537287	0.169782	-0.003409	-2.05%
4	-524.610616	-525.116675	0.164107	0.002283	1.37%
5	-603.189433	-603.690486	0.169113	-0.001933	-1.16%
6	-681.764738	-682.268579	0.166325	-0.000883	-0.53%
7	-838.907182	-839.413587	0.163761	0.000944	0.57%
8	-719.862486	-720.363828	0.168824	-0.002951	-1.78%
9	-716.309252	-716.801421	0.177997	0.016070	8.28%
10	-755.590744	-756.149505	0.111405	0.002713	2.38%
11	-603.181158	-603.689403	0.161921	0.008240	4.84%
12	-752.415179	-752.917582	0.167763	-0.003768	-2.30%
13	-697.780482	-698.945313	0.175501	1.164831	86.91%
14	-870.965188	-872.469542	0.506144	-0.002747	-0.55%
15	-639.099409	-639.603317	0.166258	-0.000278	-0.17%

Table S4. Changes in extraction reaction efficiency following phenyl group introduction at the C2 position of the hydrophobic linker region

No.	Reactants	Products	ΔG_1	ΔG_0-ΔG_1	Effect change rate (φ)
1	-444.65427	-445.133325	0.191111	-0.024661	-14.82%
2	-523.225662	-523.716765	0.179063	-0.012302	-7.38%
3	-601.801202	-602.292744	0.178624	-0.012251	-7.36%
4	-680.377017	-680.869357	0.177826	-0.011436	-6.87%
5	-758.952862	-759.444916	0.178112	-0.010932	-6.54%
6	-837.529395	-838.02026	0.179301	-0.013859	-8.38%
7	-994.680349	-995.17637	0.174145	-0.009440	-5.73%
8	-875.621549	-876.11567	0.176045	-0.010172	-6.13%
9	-872.076332	-872.55937	0.187128	0.006939	3.58%
10	-911.356147	-911.917942	0.108371	0.005747	5.04%
11	-758.950756	-759.44378	0.177142	-0.006981	-4.10%
12	-908.180718	-908.718168	0.132716	0.031279	19.07%
13	-853.532448	-854.598392	0.274388	1.065944	79.53%
14	-1026.72804	-1028.368604	0.369934	0.133463	26.51%
15	-794.869295	-795.360731	0.17873	-0.012750	-7.68%

Table S5. Changes in extraction reaction efficiency following combined hydroxyl/phenyl modification at the C2 position of the hydrophobic linker region

No.	Reactants	Products	ΔG_1	ΔG_0-ΔG_1	Effect change rate (φ)
1	-519.86174	-520.357808	0.174098	-0.007648	-4.59%
2	-598.43068	-598.91218	0.188666	-0.021905	-13.14%
3	-677.014853	-677.488425	0.196594	-0.030221	-18.16%
4	-755.585831	-756.075737	0.18026	-0.013870	-8.34%
5	-834.159274	-834.651604	0.177836	-0.010656	-6.37%
6	-912.735707	-913.22598	0.179893	-0.014451	-8.73%
7	-1069.886428	-1070.378474	0.17812	-0.013415	-8.14%
8	-950.833325	-951.322538	0.180953	-0.015080	-9.09%
9	-947.286831	-947.76978	0.187217	0.006850	3.53%
10	-986.570109	-987.132958	0.107317	0.006801	5.96%
11	-834.165371	-834.642913	0.192624	-0.022463	-13.20%
12	-983.397158	-984.538234	0.199256	-0.035261	-21.50%
13	——	——	——	——	——
14	-1101.941188	-1103.616217	0.335469	0.167928	33.36%
15	-870.078977	-870.562141	0.187002	-0.021022	-12.67%

Table S6. Changes in extraction reaction efficiency following hydroxyl group introduction at the C3 position of the hydrophobic linker region

No.	Reactants	Products	ΔG_1	ΔG_0-ΔG_1	Effect change rate (φ)
2	-367.458587	-367.961589	0.167164	-0.000403	-0.24%
3	-446.034862	-446.538028	0.167000	-0.000627	-0.38%
4	-524.610319	-525.112645	0.167840	-0.001450	-0.87%
5	-603.186873	-603.68945	0.167589	-0.000409	-0.24%
6	-681.762508	-682.266593	0.166081	-0.000639	-0.39%
7	-838.913508	-839.416411	0.167263	-0.002558	-1.55%
8	-719.854349	-720.350774	0.173741	-0.007868	-4.74%
9	-716.321372	-716.798727	0.192811	0.001256	0.65%
10	-755.592044	-756.149339	0.114118	0.112871	1.09%
11	-603.190123	-603.684996	0.175293	-0.005132	-3.02%
12	-752.414178	-752.919155	0.165189	-0.001194	-0.73%
13	-697.783846	-698.885058	0.239120	1.101212	82.16%
14	-870.959527	-872.467537	0.502488	0.000909	0.18%
15	-639.103797	-639.606108	0.167855	-0.001875	-1.13%

Table S7. Changes in extraction reaction efficiency following phenyl group introduction at the C3 position of the hydrophobic linker region

No.	Reactants	Products	ΔG_1	ΔG_0-ΔG_1	Effect change rate (φ)
2	-523.227038	-523.70682	0.190384	-0.023623	-14.17%
3	-601.801529	-602.281165	0.19053	-0.024157	-14.52%
4	-680.376892	-680.85824	0.188818	-0.022428	-13.48%
5	-758.953162	-758.953162	0.670166	-0.502986	-300.86%
6	-837.528069	-838.009541	0.188694	-0.023252	-14.05%
7	-994.679199	-994.679199	0.670166	-0.505461	-306.89%
8	-875.622528	-876.103853	0.188841	-0.022968	-13.85%
9	-872.077473	-872.56461	0.183029	0.011038	5.69%
10	-911.359736	-911.915915	0.113987	0.000131	0.11%
11	-758.951226	-759.452937	0.168455	0.001706	1.00%
12	-908.184602	-908.663855	0.190913	-0.026918	-16.41%
13	-853.535652	-854.678859	0.197125	1.143207	85.29%
14	-1026.728373	-1028.232013	0.506858	-0.003461	-0.69%
15	-794.871439	-795.40566	0.135945	0.030035	18.10%

Table S8. Changes in extraction reaction efficiency following ester group introduction at the C3 position of the hydrophobic linker region

No.	Reactants	Products	ΔG_1	ΔG_0-ΔG_1	Effect change rate (φ)
2	-441.513547	-441.996335	0.187378	-0.020617	-12.36%
3	-520.094193	-520.578766	0.185593	-0.019220	-11.55%
4	-598.672413	-599.154619	0.18796	-0.021570	-12.96%
5	-677.245999	-677.72919	0.186975	-0.019795	-11.84%
6	-755.821805	-756.30589	0.186081	-0.020639	-12.48%
7	-912.97227	-913.458383	0.184053	-0.019348	-11.75%
8	-793.915192	-794.401263	0.184095	-0.018222	-10.99%
9	-790.367543	-790.858199	0.179510	0.014557	7.50%
10	-829.650951	-830.20898	0.185062	-0.014901	1.74%
11	-677.243551	-677.728655	0.185062	-0.014901	-8.76%
12	-826.477002	-826.959182	0.187986	-0.023991	-14.63%
13	-771.840525	-772.97104	0.209817	1.130515	84.35%
14	-945.021247	-946.530928	0.500817	0.002580	0.51%
15	-713.162883	-713.644869	0.188180	-0.022200	-13.38%

Table S9. Changes in extraction reaction efficiency following combined hydroxyl/phenyl modification at the C3 position of the hydrophobic linker region

No.	Reactants	Products	ΔG_1	ΔG_0-ΔG_1	Effect change rate (φ)
2	-598.438409	-598.918664	0.189911	-0.023150	-13.88%
3	-677.011963	-677.547681	0.134448	0.031925	19.19%
4	-755.584003	-756.115495	0.138674	0.027716	16.66%
5	-834.162874	-834.644327	0.188713	-0.021533	-12.88%
6	-912.737887	-913.221893	0.18616	-0.020718	-12.52%
7	-1069.89125	-1070.371608	0.189808	-0.025103	-15.24%
8	-950.825905	-951.367616	0.128455	0.037418	22.56%
9	-947.285914	-987.125684	0.182141	0.011926	6.15%
10	-986.568743	-947.834989	0.113225	0.000893	0.78%
11	-834.157363	-834.695953	0.131576	0.038585	22.68%
12	-983.388992	-983.879466	0.179692	-0.015697	-9.57%
13	-928.747821	-929.813408	0.274745	1.065587	79.50%
14	-1097.736294	-1099.203529	0.543263	-0.039866	-7.92%
15	-870.078638	-870.554457	0.194347	-0.028367	-17.09%

Table S10. Changes in extraction reaction efficiency following hydroxyl group introduction at the C4 position of the hydrophobic linker region

No.	Reactants	Products	ΔG_1	ΔG_0-ΔG_1	Effect change rate (φ)
2	-367.453903	-367.956675	0.167394	-0.000633	-0.38%
3	-446.033974	-446.538189	0.165951	0.000422	0.25%
4	-524.610906	-525.114342	0.16673	-0.00034	-0.20%
5	-603.184237	-603.688284	0.166119	0.001061	0.63%
6	-681.764738	-682.26365	0.171254	-0.005812	-3.51%
7	-838.910698	-839.415421	0.165443	-0.000738	-0.45%
8	-719.857313	-720.360457	0.167022	-0.001149	-0.69%
9	-716.310302	-716.801265	0.179203	0.014864	7.66%
10	-755.590744	-756.148978	0.111932	0.002186	1.92%
11	-603.182978	-603.684963	0.168181	0.00198	1.16%
12	-752.414574	-752.918473	0.166267	-0.002272	-1.39%
13	-697.777271	-698.882803	0.2348	1.105532	82.48%
14	-870.963743	-873.793926	-0.819685	1.323082	262.83%
15	-639.102265	-639.605185	0.167246	-0.001266	-0.76%

Table S11. Changes in extraction reaction efficiency following phenyl group introduction at the C4 position of the hydrophobic linker region

No.	Reactants	Products	ΔG_1	ΔG_0-ΔG_1	Effect change rate (φ)
2	-523.22992	-523.704902	0.195184	-0.028423	-17.04%
3	-601.801125	-602.279569	0.191722	-0.025349	-15.24%
4	-680.378148	-680.855346	0.192968	-0.026578	-15.97%
5	-758.952483	-759.431434	0.191215	-0.024035	-14.38%
6	-837.528584	-838.007754	0.190996	-0.025554	-15.45%
7	-994.679107	-995.156216	0.193057	-0.028352	-17.21%
8	-875.622696	-876.104817	0.188045	-0.022172	-13.37%
9	-872.076374	-872.564089	0.182451	0.011616	5.99%
10	-911.359618	-911.915101	0.114683	-0.000565	-0.50%
11	-758.949506	-759.43158	0.188092	-0.017931	-10.54%
12	-908.182842	-908.655441	0.197567	-0.033572	-20.47%
13	-815.401657	-816.565856	0.176133	1.164199	86.86%
14	-1026.727568	-1029.636284	-0.898218	1.401615	278.43%
15	-794.869275	-795.362648	0.176793	-0.010813	-6.51%

Table S12. Changes in extraction reaction efficiency following ester group introduction at the C4 position of the hydrophobic linker region

No.	Reactants	Products	ΔG_1	ΔG_0-ΔG_1	Effect change rate (φ)
2	——	——	——	——	——
3	-520.094648	-520.573405	0.191409	-0.025036	-15.05%
4	-598.669738	-599.147238	0.192666	-0.026276	-15.79%
5	-677.238264	-677.724034	0.184396	-0.017216	-10.30%
6	-755.820516	-756.301512	0.18917	-0.023728	-14.34%
7	-912.973109	-913.453054	0.190221	-0.025516	-15.49%
8	-793.916141	-794.398908	0.187399	-0.021526	-12.98%
9	-790.368261	-790.859816	0.178611	0.015456	7.96%
10	-829.652245	-830.20815	0.114261	-0.000143	-0.13%
11	-677.243112	-677.724315	0.188963	-0.018802	-11.05%
12	-826.475083	-826.995043	0.150206	0.013789	8.41%
13	-771.84218	-772.870933	0.311579	1.028753	76.75%
14	-945.015526	-946.631405	0.394619	0.108778	21.61%
15	-713.162726	-713.640339	0.192553	-0.026573	-16.01%

Table S13. Changes in extraction reaction efficiency following combined hydroxyl/phenyl modification at the C4 position of the hydrophobic linker region

No.	Reactants	Products	ΔG_1	ΔG_0-ΔG_1	Effect change rate (φ)
2	-598.438528	-598.917194	0.1915	-0.024739	-14.84%
3	-677.012208	-677.492636	0.189738	-0.023365	-14.04%
4	-755.588628	-756.06666	0.192134	-0.025744	-15.47%
5	-834.162526	-834.643212	0.18948	-0.022300	-13.34%
6	-912.738292	-913.219096	0.189362	-0.023920	-14.46%
7	-1069.890135	-1070.370336	0.189965	-0.025260	-15.34%
8	-950.834515	-951.316398	0.188283	-0.022410	-13.51%
9	-947.288154	-947.774298	0.184022	0.010045	5.18%
10	-986.571009	-987.122749	0.118426	-0.004308	-3.78%
11	-834.161628	-834.643331	0.188463	-0.018302	-10.76%
12	-983.38873	-982.48274	1.576156	-1.412161	-861.10%
13	-890.627434	-891.689141	0.278625	1.061707	79.21%
14	-1101.941022	-1104.796534	-0.845014	1.348411	267.86%
15	-870.080337	-870.559323	0.19118	-0.025200	-15.18%

Table S14. Gibbs free energies of CO_2, H_2O, and HCO_3^- in the solvent recovery environments

Recovery reaction environment	CO_2(Hartree)	H_2O(Hartree)	HCO_3^-(Hartree)
ethanol	-188.592451	-76.412536	-264.332452
water	-188.59256	-76.412853	-264.332452

Table S15. Gibbs free energies of the recovery reactions of CO_2-switchable solvent molecules in the solvent recovery environments

Original molecule	Reaction energy		Modifying molecules	Reaction energy	
	Ethanol	Water		Ethanol	Water
3	-0.168657	-0.169057	3-3-OH-Ph	-0.135801	-0.198147
4	-0.168408	-0.168766	3-4-OH-Ph	-0.140078	-0.140779
8	-0.168769	-0.169126	3-8-C-OH-Ph	-0.131320	-0.131828
11	-0.171389	-0.172047	3-11-C-OH-Ph	-0.131029	-0.202234
12	-0.169025	-0.169025	2-12-C-Ph	-0.138118	-0.136809
13	-0.289730	-0.290700	2-13-C-oh	-0.148973	-0.145701
			2-13-C-Ph	-0.289011	-0.28757
			3-13-C-Ph	-0.204663	-0.202234
			3-13-C-COO	-0.236593	-0.215961
			3-13-C-OH-Ph	-0.287478	-0.285386
			3-13-C-OH	-0.247356	-0.248939
			3-13-C	-0.288276	-0.280691
			4-13-C-oh	-0.233351	-0.259549
			4-13-C-Ph	-0.173483	-0.174372
			4-13-C-COO	-0.310876	-0.323004
			4-13-C-oh-Ph	-0.27554	-0.276415
14	-0.508288	-522.247698	2-14-C-Ph	-0.478365	-0.478365
			2-14-C-oh-Ph	-0.315915	-0.315915
			4-14-C-oh	-0.508683	-0.509965
			4-14-C-Ph	-0.507874	-0.509201
			4-14-C-	-0.530275	-0.532384
			4-14-C-oh-Ph	-0.477434	-0.479753
15	-0.168117	-0.169057	3-15-C-Ph	-0.137785	-0.203124

(Note: Molecular labels are coded as modification site–parent molecule number–modification strategy. Abbreviations: -C, C_8-equivalent hydrophobic-tail modification; -OH, hydroxyl-group modification; -Ph, phenyl-group modification; and -COO, ester-group modification.)

Table S16. Predicted values for 24 toxicity endpoints of CO_2-switchable solvent molecules

Molecular name	Toxicity(abbreviation)																									
	hERG Blockers	hERG Blockers (10um)	DILI	AMES Toxicity	Rat Oral Acute Toxicity	FDAMDD	Skin Sensitization	Carcinogenicity	Eye Corrosion	Eye Irritation	Respiratory	Human Hepatotoxicity	Drug-induced Nephrotoxicity	Drug-induced Neurotoxicity	Ototoxicity	Hematotoxicity	Genotoxicity	RPMI-8226 Immunitoxicity	A549 Cytotoxicity	Hek293 Cytotoxicity	BCF	IGC50	LC50DM	LC50FM
	hERG Blockers	hERG (10um)	DILI	AMES	Rat LD50	FDAMDD	Skin Sens.	Carc.	Eye Corr.	Eye Irrit.	Resp.	H-HT	DI-Neph	DI-Neuro	Ototox.	Hematotox.	Genotox.	RPMI-8226	A549	HEK293	BCF	IGC50	LC50DM	LC50FM
3	0.639	0.741	0.095	0.306	0.653	0.240	0.978	0.424	0.997	0.941	0.962	0.686	0.735	0.359	0.403	0.650	0.003	0.064	0.144	0.130	1.026	3.040	4.275	3.617
4	0.652	0.772	0.111	0.277	0.616	0.241	0.983	0.445	0.996	0.950	0.964	0.688	0.663	0.350	0.405	0.567	0.002	0.064	0.191	0.129	2.012	3.978	4.860	4.565
8	0.342	0.647	0.049	0.256	0.439	0.533	0.894	0.262	0.979	0.879	0.895	0.563	0.763	0.373	0.314	0.395	0.003	0.072	0.114	0.177	1.057	3.051	4.177	3.740
11	0.586	0.719	0.041	0.415	0.712	0.255	0.992	0.298	0.993	0.888	0.997	0.344	0.947	0.269	0.496	0.257	0.303	0.034	0.030	0.126	0.200	2.039	3.240	2.338
12	0.155	0.457	0.265	0.570	0.499	0.318	0.575	0.247	0.979	0.982	0.921	0.734	0.724	0.723	0.362	0.736	0.057	0.039	0.044	0.042	0.081	1.339	2.254	1.365
13	0.831	0.719	0.099	0.605	0.704	0.316	0.987	0.618	0.987	0.328	0.947	0.802	0.927	0.498	0.519	0.728	0.054	0.093	0.063	0.130	0.211	2.139	3.337	2.540
14	0.703	0.733	0.068	0.701	0.355	0.245	0.997	0.661	0.991	0.034	0.846	0.898	0.954	0.653	0.606	0.624	0.016	0.154	0.075	0.105	0.506	2.162	3.328	2.432
15	0.220	0.479	0.153	0.520	0.338	0.119	0.948	0.654	0.988	0.982	0.866	0.645	0.644	0.399	0.422	0.515	0.049	0.054	0.039	0.078	0.068	1.399	2.542	1.761
2-12-Ph	0.552	0.816	0.067	0.243	0.369	0.239	0.754	0.136	0.402	0.819	0.669	0.553	0.477	0.646	0.125	0.528	0.001	0.053	0.304	0.389	1.854	4.040	5.187	4.679
2-13-Ph	0.874	0.897	0.072	0.207	0.550	0.383	0.923	0.332	0.793	0.335	0.926	0.811	0.761	0.552	0.469	0.731	0.001	0.079	0.548	0.380	1.877	4.208	5.061	4.747
2-13-OH	0.809	0.789	0.021	0.153	0.427	0.215	0.991	0.363	0.992	0.462	0.966	0.790	0.562	0.619	0.480	0.767	0.001	0.078	0.342	0.202	1.419	3.633	4.864	4.279
2-14-Ph	0.860	0.917	0.027	0.349	0.242	0.248	0.997	0.252	0.968	0.115	0.913	0.889	0.923	0.688	0.286	0.789	0.000	0.106	0.293	0.379	2.250	4.389	5.095	4.978
2-14-OH-Ph	0.847	0.861	0.018	0.269	0.194	0.282	0.998	0.252	0.991	0.187	0.931	0.865	0.850	0.708	0.248	0.857	0.000	0.096	0.288	0.311	1.889	3.972	4.989	4.606
3-11-OH-Ph	0.811	0.857	0.020	0.192	0.381	0.460	0.995	0.092	0.932	0.681	0.996	0.413	0.902	0.294	0.212	0.677	0.021	0.034	0.148	0.289	1.894	3.946	4.861	4.504
3-13-Ph	0.957	0.925	0.120	0.279	0.749	0.639	0.982	0.348	0.277	0.007	0.979	0.939	0.896	0.914	0.535	0.929	0.008	0.146	0.519	0.477	1.970	4.089	5.016	4.657
3-13-C8	0.933	0.902	0.055	0.236	0.763	0.377	0.997	0.335	0.990	0.103	0.990	0.912	0.725	0.847	0.527	0.885	0.000	0.150	0.406	0.239	1.589	3.747	4.932	4.361
3-13-COO	0.900	0.857	0.185	0.309	0.697	0.400	0.988	0.657	0.495	0.005	0.944	0.929	0.873	0.871	0.525	0.899	0.020	0.161	0.205	0.281	0.755	3.311	4.682	4.182

Continuous Table S16. Predicted values for 24 toxicity endpoints of CO_2-switchable solvent molecules

Molecular name	Toxicity(abbreviation)																							
	hERG Blockers	hERG Blockers (10um)	DILI	AMES Toxicity	Rat Oral Acute Toxicity	FDAMDD	Skin Sensitization	Carcinogenicity	Eye Corrosion	Eye Irritation	Respiratory	Human Hepatotoxicity	Drug-induced Nephrotoxicity	Drug-induced Neurotoxicity	Ototoxicity	Hematotoxicity	Genotoxicity	RPMI-8226 Immunitoxicity	A549 Cytotoxicity	Hek293 Cytotoxicity	BCF	IGC50	LC50DM	LC50FM
	hERG Blockers	hERG (10um)	DILI	AMES	Rat LD50	FDAMDD	Skin Sens.	Carc.	Eye Corr.	Eye Irrit.	Resp.	H-HT	DI-Neph	DI-Neuro	Ototox.	Hematotox.	Genotox.	RPMI-8226	A549	HEK293	BCF	IGC50	LC50DM	LC50FM
3-13-OH	0.841	0.875	0.102	0.283	0.678	0.485	0.954	0.519	0.802	0.039	0.965	0.912	0.765	0.950	0.545	0.888	0.011	0.111	0.234	0.274	0.564	3.175	4.584	3.807
3-13-OH-Ph	0.884	0.882	0.109	0.216	0.539	0.630	0.974	0.366	0.253	0.010	0.973	0.922	0.848	0.885	0.497	0.924	0.026	0.106	0.343	0.373	1.352	3.693	4.756	4.290
3-15-Ph	0.822	0.812	0.078	0.237	0.332	0.188	0.981	0.343	0.895	0.875	0.930	0.737	0.586	0.527	0.370	0.863	0.002	0.065	0.294	0.408	2.014	3.990	5.044	4.588
3-3-OH-Ph	0.518	0.742	0.034	0.190	0.370	0.598	0.952	0.250	0.530	0.308	0.927	0.712	0.885	0.473	0.346	0.703	0.063	0.049	0.102	0.182	0.419	2.935	4.089	3.419
3-4-OH-Ph	0.610	0.774	0.040	0.151	0.310	0.597	0.977	0.192	0.829	0.599	0.950	0.685	0.782	0.455	0.263	0.677	0.006	0.047	0.189	0.193	1.801	3.868	4.882	4.484
3-8-OH-Ph	0.726	0.805	0.023	0.100	0.200	0.714	0.892	0.078	0.554	0.478	0.950	0.601	0.610	0.519	0.221	0.668	0.001	0.048	0.340	0.402	2.016	4.189	5.065	4.759
4-13-Ph	0.955	0.916	0.115	0.323	0.837	0.693	0.992	0.561	0.326	0.003	0.982	0.956	0.883	0.938	0.672	0.924	0.062	0.203	0.396	0.475	1.833	4.145	5.277	4.797
4-13-COO	0.877	0.841	0.150	0.257	0.707	0.518	0.986	0.599	0.806	0.028	0.946	0.889	0.741	0.847	0.398	0.893	0.011	0.122	0.095	0.146	0.851	3.201	4.409	3.888
4-13-OH	0.927	0.872	0.073	0.200	0.586	0.606	0.993	0.346	0.799	0.009	0.987	0.939	0.789	0.962	0.562	0.965	0.009	0.128	0.220	0.300	0.758	3.168	4.352	3.758
4-13-OH-Ph	0.941	0.889	0.094	0.386	0.763	0.757	0.987	0.478	0.261	0.002	0.982	0.952	0.885	0.940	0.659	0.924	0.056	0.194	0.403	0.411	1.226	3.519	4.942	4.192
4-14-Ph	0.964	0.951	0.020	0.495	0.244	0.297	0.999	0.170	0.967	0.018	0.849	0.937	0.901	0.766	0.379	0.919	0.000	0.181	0.371	0.619	2.277	4.287	5.120	4.919
4-14-COO	0.788	0.845	0.037	0.476	0.142	0.143	0.997	0.449	0.991	0.129	0.644	0.784	0.806	0.512	0.212	0.856	0.000	0.132	0.014	0.112	0.982	3.359	4.546	4.165
4-14-OH	0.857	0.882	0.013	0.340	0.078	0.222	0.999	0.168	0.986	0.065	0.923	0.894	0.799	0.890	0.291	0.902	0.000	0.118	0.080	0.227	0.893	3.266	4.550	4.015
4-14-OH-Ph	0.888	0.908	0.008	0.410	0.100	0.277	0.998	0.184	0.982	0.045	0.850	0.894	0.917	0.712	0.264	0.938	0.000	0.116	0.165	0.391	1.602	3.722	4.962	4.457

Table S17. Structure guide and nomenclature of the selected modified CSS candidates.

Molecular label	Parent No.	Modification site	C8 standardization	Functional-group modification
3-3-OH-Ph	3	C3	No	Hydroxyl + phenyl (OH + Ph)
3-4-OH-Ph	4	C3	No	Hydroxyl + phenyl (OH + Ph)
3-8-C-OH-Ph	8	C3	Yes	Hydroxyl + phenyl (OH + Ph)
3-11-C-OH-Ph	11	C3	Yes	Hydroxyl + phenyl (OH + Ph)
2-12-C-Ph	12	C2	Yes	Phenyl (Ph)
2-13-C-OH	13	C2	Yes	Hydroxyl (OH)
2-13-C-Ph	13	C2	Yes	Phenyl (Ph)
3-13-C-OH	13	C3	Yes	Hydroxyl (OH)
3-13-C-Ph	13	C3	Yes	Phenyl (Ph)
3-13-C-COO	13	C3	Yes	Ester (COO)
3-13-C-OH-Ph	13	C3	Yes	Hydroxyl + phenyl (OH + Ph)
13-C	13	N/A	Yes	None
4-13-C-OH	13	C4	Yes	Hydroxyl (OH)
4-13-C-Ph	13	C4	Yes	Phenyl (Ph)
4-13-C-COO	13	C4	Yes	Ester (COO)
4-13-C-OH-Ph	13	C4	Yes	Hydroxyl + phenyl (OH + Ph)
2-14-C-Ph	14	C2	Yes	Phenyl (Ph)
2-14-C-OH-Ph	14	C2	Yes	Hydroxyl + phenyl (OH + Ph)
4-14-C-OH	14	C4	Yes	Hydroxyl (OH)
4-14-C-Ph	14	C4	Yes	Phenyl (Ph)
4-14-C-COO	14	C4	Yes	Ester (COO)
4-14-C-OH-Ph	14	C4	Yes	Hydroxyl + phenyl (OH + Ph)
3-15-C-Ph	15	C3	Yes	Phenyl (Ph)
